# Robust twin-field quantum key distribution through sending or not sending

**DOI:** 10.1093/nsr/nwac186

**Published:** 2022-09-19

**Authors:** Cong Jiang, Zong-Wen Yu, Xiao-Long Hu, Xiang-Bin Wang

**Affiliations:** Jinan Institute of Quantum Technology, Jinan 250101, China; State Key Laboratory of Low Dimensional Quantum Physics, Department of Physics, Tsinghua University, Beijing 100084, China; Data Communication Science and Technology Research Institute, Beijing 100191, China; School of Physics, State Key Laboratory of Optoelectronic Materials and Technologies, Sun Yat-sen University, Guangzhou 510275, China; Jinan Institute of Quantum Technology, Jinan 250101, China; State Key Laboratory of Low Dimensional Quantum Physics, Department of Physics, Tsinghua University, Beijing 100084, China; Shanghai Branch, CAS Center for Excellence and Synergetic Innovation Center in Quantum Information and Quantum Physics, University of Science and Technology of China, Shanghai 201315, China; Shenzhen Institute for Quantum Science and Engineering, and Physics Department, Southern University of Science and Technology, Shenzhen 518055, China; Frontier Science Center for Quantum Information, Beijing 100084, China

**Keywords:** quantum information, quantum key distribution, sending or not sending, twin field, source error

## Abstract

The sending-or-not-sending (SNS) protocol is one of the most major variants of the twin-field (TF) quantum key distribution (QKD) protocol and has been realized in a 511-km field fiber, the farthest field experiment to date. In practice, however, all decoy-state methods have unavoidable source errors, and the source errors may be non-random, which compromises the security condition of the existing TF-QKD protocols. In this study, we present a general approach for efficiently calculating the SNS protocol’s secure key rate with source errors, by establishing the equivalent protocols through virtual attenuation and the tagged model. This makes the first result for TF QKD in practice where source intensity cannot be controlled exactly. Our method can be combined with the two-way classical communication method such as active odd-parity pairing to further improve the key rate. The numerical results show that if the intensity error is within a few percent, the key rate and secure distance only decrease marginally. The key rate of the recent SNS experiment in the 511-km field fiber is still positive using our method presented here, even if there is a $\pm 9.5\%$ intensity fluctuation. This shows that the SNS protocol is robust against source errors.

## Teaser

Robust twin-field QKD protocol through sending-or-not-sending is proposed, producing secure bits efficiently with intensity errors of source pulses.

## INTRODUCTION

Since the proposal of the twin-field (TF) quantum key distribution (QKD) [1], the longest distance record of QKD has been constantly and rapidly refreshed in recent years [2–9]. The upper bounds of the key rate for previous protocols, such as the BB84 protocol [10–15] and the measurement-device-independent (MDI) QKD protocol [16–22] are limited to the linear scale of channel transmittance, also known as the PLOB bound [23]. Based on single-photon interference, the TF QKD can raise the key rate from a linear scale to a square-root scale of channel transmittance and break the PLOB bound.

As one of the most important variants of TF QKD [24–27], the sending-or-not-sending (SNS) protocol [24] has been extensively studied in theories [28–32] and experiments [2,6–9,33]. Specifically, the 511-km field experiment [8] was done by applying the SNS protocol with the actively odd-parity pairing (AOPP) method, and the 605-km laboratory experiment [9] was done by applying the SNS protocol with the standard two-way classical communication (TWCC) method. The 511-km experiment is the farthest field experiment to date.

Given its great progress [34,35] in both theory and technology, more and more studies of QKD are pointing to real-world applications with various technology limitations.

The TF QKD has typically used with weak coherent state (WCS) sources. The decoy-state method must be used to ensure the security of TF QKD with WCS sources. The intensities of WCS sources are always assumed to be stable in the entire protocol, which is not the fact in experiments where source errors are unavoidable. The source errors, in particular, are not always random. For example, the intensities of the sources can slowly shift over time and are affected by temperature changes in the environment.

Although there are results for decoy-state analysis with source errors for the BB84 and MDI-QKD protocols [36,37], they do not solve the problem for the SNS protocol where encoding is done by the vacuum and single-photon states themselves. To calculate the key rate, we need a way to estimate the single-photon phase-flip error rate. It is not a trivial task because the single-photon state in the *X* basis is now different from that in the *Z* basis. We solve this problem by virtual attenuation and then apply the existing theory for decoy-state analysis with intensity fluctuation.

The work is organized as follows. First, we introduce the real and virtual protocols and demonstrate their equivalence. This shows how to efficiently calculate the secure key rate in a practical application where intensities cannot be controlled exactly. Then we present some numerical results that compare the key rates of the SNS protocol with different degrees of source errors.

## THE SECURITY OF THE SNS PROTOCOL WITH SOURCE ERRORS

For ease of presentation, here we introduce the SNS protocol through the four-intensity model. The scientific content is the same as in the earlier literature.

We take the four-intensity SNS protocol as an example to show the security proof. There are four sources with different intensities on Alice’s and Bob’s sides. We denote Alice’s sources by *a*_0_, *a*_1_, *a*_2_ and *a_z_*, and Bob’s sources by *b*_0_, *b*_1_, *b*_2_ and *b_z_*, where *a*_0_ and *b*_0_ are the vacuum sources. WCS sources are typically used to perform the SNS protocol. If the sources are stable, the intensities of sources *a_l_* and *b_r_* are $\mu _{a_l}$ and $\mu _{b_r}$, respectively, for *l*, *r* = 0, 1, 2, *z*, where $\mu _{a_0}=\mu _{b_0}=0$. However, in practice, the sources are always unstable and the intensities are different in different time windows. The intensities of the sources in the *i*th time window are denoted $\mu _{a_l}^i$ and $\mu _{b_r}^i$, where


(1) \begin{eqnarray*} \mu _{a_l}^i=(1+\delta _{a_l}^i)\mu _{a_l}, \qquad \mu _{b_r}^i=(1+\delta _{b_r}^i)\mu _{b_r} \nonumber\\ \end{eqnarray*}

and $|\delta _{a_l}^i|\le \delta _{a_l},|\delta _{b_r}^i|\le \delta _{b_r}$. We assume that $\delta _{a_l}$ and $\delta _{b_r}$ are known values in the protocol. The lower and upper bounds of a physical quantity are represented by superscripts *L* and *U*, respectively. For instance, $\mu _{a_1}^U$ is the upper bound of the intensity of source *a*_1_, and we have $\mu _{a_1}^U=(1+\delta _{a_1})\mu _{a_1}$.

### The real protocol

Alice (Bob) randomly decides whether the *i*th time window is a decoy window or a signal window with probabilities $1-p_{a_z}$ and $p_{a_z}$ ($1-p_{b_z}$ and $p_{b_z}$). If it is a decoy window, Alice (Bob) randomly chooses sources *a*_0_, *a*_1_, *a*_2_ (*b*_0_, *b*_1_, *b*_2_) with probabilities $p_{a_0}=1-p_{a_1}-p_{a_2},p_{a_1}, p_{a_2}$ ($p_{b_0}=1-p_{b_1}-p_{b_2}, p_{b_1}, p_{b_2}$). Since the sources are unstable, Alice (Bob) actually prepares a WCS pulse in state $|e^{\theta _{a_l}^i}\sqrt{\mu _{a_l}^i}\rangle$ ($|e^{\theta _{b_r}^i}\sqrt{\mu _{b_r}^i}\rangle$) if source *a_l_* (*b_r_*) is chosen for *l*, *r* = 1, 2, where $\theta _{a_l}$ and $\theta _{b_r}$ are random in [0, 2π). The imaginary unit is represented here by the symbol ı. If it is a signal window, Alice (Bob) randomly chooses sources *a*_0_, *a_z_* (*b*_0_, *b_z_*) with probabilities 1 − ε_*a*_, ε_*a*_ (1 − ε_*b*_, ε_*b*_). Alice (Bob) actually prepares a phase-randomized WCS pulse with intensity $\mu _{a_z}^i$ ($\mu _{b_z}^i$) if source *a_z_* (*b_z_*) is chosen. Clearly, if Alice (Bob) commits to a signal window at time *i*, Alice’s (Bob’s) choice of source *a_z_* (*b_z_*) indicates a decision of *sending* whereas the choice of source *a*_0_ (*b*_0_) is a decision of *not sending* in the SNS protocol.

*They* (Alice and Bob) send the prepared pulse pair to Charlie, who is assumed to perform interferometric measurements on the received pulse pair. Then Charlie announces the measurement results to *them*. If only one detector clicks, *they* would take it as a *one-detector heralded event*. After *they* repeat the above process *N* times and Charlie announces all measurement results, *they* acquire a series of data. Then *they* perform the data post-processing, including post-selection of events in *X* windows and final key distillation.

*They* first announce the type of time window they choose, i.e. whether it is a decoy window or a signal window. For a time window that both of *them* decide is a signal window, it is a *Z* window. The one-detector heralded events in *Z* windows are effective events, and the corresponding bits of those effective events form the *n_t_*-bit raw key strings, which are used to extract the final keys. We also define the $\tilde{Z}$ window as a *Z* window when sources {*a*_0_, *b_z_*} or {*a_z_*, *b*_0_} are used, i.e. a *Z* window when one side decides sending and the other side decides not sending. The untagged pulses in the *Z* windows are that Alice decides not sending and Bob actually sends out a single-photon pulse or Bob decides not sending and Alice actually sends out a single-photon pulse, i.e. the single-photon pulses in the $\tilde{Z}$ windows. The untagged bits are the corresponding bits of those untagged pulses that cause effective events. Except the *Z* windows, *they* announce the intensities of the pulses in each window, and for time windows that both of *them* decide sending out a pulse of sources *a*_1_ and *b*_1_, respectively, *they* also announce the phases of the pulse pairs. For a time window that both of *them* decide sending out a pulse of sources *a*_1_ and *b*_1_, respectively, and their phases satisfy the post-selection criteria [24], it is defined as an *X* window. The one-detector heralded events in *X* windows are effective events. The effective events in *X* windows are used to estimate the phase-flip error rate.

According to Hu and Jiang [30], if the sources are stable, and source *a_z_* (*b_z_*) always emits a state of $\rho _{a_z}=\sum _{k=0} q_A^k |k\rangle \langle k|$, ($\rho _{b_z}=\sum _{k=0} q_B^k |k\rangle \langle k|$), we can use the following constraint for the source for security:


(2) \begin{eqnarray*} \frac{\mu _{a_1}}{\mu _{b_1}}=\frac{\epsilon _a(1-\epsilon _b)q_A^1}{\epsilon _b(1-\epsilon _a)q_B^1}. \end{eqnarray*}

Here |*k*〉〈*k*| represents a *k*-photon Fock state. In particular, when using a phase randomized WCS source with intensity $\mu _{a_z}$ ($\mu _{b_z}$) for source *a_z_* (*b_z_*), $q_A^1, q_B^1$ in (2) can be replaced by $\mu _{a_z}e^{-\mu _{a_z}}, \mu _{b_z}e^{-\mu _{b_z}}$. Note that, if the constraint above is respected, the density matrix of the single-photon state in $\tilde{Z}$ windows is identical to that of the *X* basis and hence we can faithfully verify the phase-flip error rate of untagged bits by observing events in *X* windows.

Given stable sources with the source parameters known, the SNS protocol is still secure and efficient even if (2) does not hold. Most straightforwardly, we can post-select the sending probabilities ε_*a*_, ε_*b*_ by randomly deleting some of the time windows [6], i.e. to make (2) hold by post-deletion. (Post-deleting a time window means disregarding the event happening at that time window.) Although the amount of data is reduced by the post-deletion, the key rate will essentially be unchanged if the original bias between the two sides of (2) is not too large, because, after post-deletion, the error rate of raw keys is also decreased.

The more practical situation is that we do not know the exact intensities of the sources of the *Z* windows but we know the bound values of intensities for each source of *Z* window. In such a case, we can use the tagged model: replace the intensities of (2) by a lower bound value and make (2) hold.

Most generally, the source intensities of *X* windows are also unstable. Say, in practice, that none of the sources are stable, and that the intensity errors may be different from time to time and not necessarily random. In such a case, Eve can treat different time windows differently; thus, we cannot treat the *X* windows as a whole, but have to deal with each time window separately. The intensities of sources *a*_1_, *b*_1_ in different time windows are different, which means that, in general,


(3) \begin{eqnarray*} \frac{\mu _{a_1}^i}{\mu _{b_1}^i}\ne \frac{\mu _{a_1}^j}{\mu _{b_1}^j} \end{eqnarray*}

if *i* ≠ *j*. When (2) holds, the bit-flip error rate of *X* windows can be used to estimate the phase-flip error rate of the untagged bits in *Z* windows. But, due to source errors, (2) cannot hold for all *X* windows, so we cannot estimate the phase-flip error rate of the untagged bits according to the data in *X* windows. The situation is different for the decoy-state BB84 protocol with source errors [36], or the decoy-state MDI QKD [16,22] where the source errors do not change the encoding states in *X* windows or *Z* windows, it affects the decoy-state analysis only [36,37]. In this work, we solve this problem based on the SNS protocol and make the TF QKD robust with imperfect real set-ups.

### Main idea: quantum security from virtual attenuation

The state preparation stage of the real protocol can be regarded as follows. At every time window *i*, Alice (Bob) has four candidate states emitted from the four sources *a*_0_, *a*_1_, *a*_2_, *a_z_* (*b*_0_, *b*_1_, *b*_2_, *b_z_*). In particular, the intensities of the coherent states emitted by sources *a_l_*, *b_r_* are $\mu _{a_l}^i, \mu _{b_r}^i$ for *l*, *r* = 1, 2, *z*, respectively. Sources *a*_0_, *b*_0_ emit vacuum only. Alice (Bob) will choose one state from the four candidate states for time window *i*, by the probabilities stated earlier in the real protocol.

Our first major idea is mapping by (virtual) attenuation [38]. We regard the candidate states above as the attenuation outcome from virtual sources $a_0, a_1^\prime , a_2^\prime , a_z^\prime$ ($b_0,b_1^\prime , b_2^\prime , b_z^\prime$) and the intensity of the WCS pulse from virtual source $a_1^\prime$ ($b_1^\prime$) is fixed to $\mu _{a_1}^U$ ($\mu _{b_1}^U$) exactly at any time window *i*. This naturally means that the transmittance $\eta _A^i={(1+\delta _{a_1}^i)}/{(1+\delta _{a_1})}$ [$\eta _B^i={(1+\delta _{b_1}^i)}/{(1+\delta _{b_1})}$] in the virtual attenuation of the *i*th time window, and hence the intensities of the WCS pulses from virtual sources $a_2^\prime ,a_z^\prime$ ($b_2^\prime ,b_z^\prime$) are $\mu _{a_2}^{\prime i}= \mu _{a_2}^{i}/\eta _A^i, \mu _{a_z}^{\prime i}= \mu _{a_z}^{i}/\eta _A^i$, ($\mu _{b_2}^{\prime i}= \mu _{b_2}^{i}/\eta _B^i,\mu _{b_z}^{\prime i}= \mu _{b_z}^{i}/\eta _B^i$), respectively. For simplicity, we just call the SNS protocol using virtual (real) sources above the virtual protocol (real protocol). Surely, the real protocol above is secure provided that the virtual protocol above is secure. This holds even though Eve knew the exact values of the intensity fluctuation for each source in every time window *i*. Or, equivalently, even though Eve knew the exact values of $\eta _a^i$, $\eta _b^i$. If Eve can use scheme $\mathcal {G}$ to attack the real protocol, Eve can also attack the virtual protocol by first taking attenuation $\lbrace \eta _A^i,\eta _B^i\rbrace$ to pulses in the virtual protocol and then using attacking scheme $\mathcal {G}$. Surely, we can obtain the secure key rate for the real protocol above by calculating the secure key rate of the virtual protocol, because the two protocols cause no difference to the outside lab.

Our second major idea is to treat the intensity fluctuation for states of the *Z* windows using the tagged model. Consider the key rate calculation for our virtual protocol above. In that protocol, at time window *i*, the intensity of the pulse from virtual source $a_z^\prime$ ($b_z^\prime$) is $\mu _{a_z}^\prime$ ($\mu _{b_z}^\prime$). This in general does not respect (2). However, in $\tilde{Z}$ windows, there does exist a subset of single-photon pulses that are indistinguishable from the single photons in *X* windows, and there does exist an efficient method to verify the lower bound of the size of this subset. Thus, the key rate can be effectively calculated.

The third major idea here is to verify the lower bound values of the untagged bits in *Z* windows and their corresponding upper bound value of the phase-flip error rate, by applying the existing decoy-state method with source errors [36]. Note that in the virtual protocol above, the intensities of the stronger decoy pulses are not stable. According to the existing theory, we only need to use the lower bound values of the photon number distribution coefficients to obtain the worst-case result. With these, the final key rate can be calculated. Furthermore, we can apply the TWCC method such as AOPP to improve the key rate [31,32].

### Virtual protocol 1

In the *i*th time window of virtual protocol 1, the pulses emitted by sources *a*_1_, *a*_2_, *a_z_* (*b*_1_, *b*_2_, *b_z_*) are different from those of the real protocol. To avoid confusion, we use the notation $a_0, a_1^\prime , a_2^\prime , a_z^\prime$ ($b_0, b_1^\prime , b_2^\prime , b_z^\prime$) for Alice’s (Bob’s) sources in virtual protocol 1.

In the *i*th time window, Alice (Bob) randomly decides it is a decoy window or a signal window with probabilities $1-p_{a_z}$ and $p_{a_z}$ ($1-p_{b_z}$ and $p_{b_z}$). If it is a decoy window, Alice (Bob) randomly chooses sources $a_0,a_1^\prime ,a_2^\prime$ ($b_0,b_1^\prime ,b_2^\prime$) with probabilities $p_{a_0}=1-p_{a_1}-p_{a_2},p_{a_1},p_{a_2}$ ($p_{b_0}=1-p_{b_1}-p_{b_2},p_{b_1},p_{b_2}$). If source $a_1^\prime$ ($b_1^\prime$) is chosen, Alice (Bob) actually prepares a WCS pulse in state $|e^{\theta _{a_1}^i}\sqrt{\mu _{a_1}^U}\rangle$ ($|e^{\theta _{b_1}^i}\sqrt{\mu _{b_1}^U}\rangle$), where $\mu _{a_1}^U=(1+\delta _{a_1})\mu _{a_1}$ and $\mu _{b_1}^U=(1+\delta _{b_1})\mu _{b_1}$. If source $a_2^\prime$ ($b_2^\prime$) is chosen, Alice (Bob) actually prepares a WCS pulse in state $|e^{\theta _{a_2}^i}\sqrt{\mu _{a_2}^{\prime i}}\rangle$ ($|e^{\theta _{b_2}^{i}}\sqrt{\mu _{b_2}^{\prime i}}\rangle$), where


(4) \begin{eqnarray*} \mu _{a_2}^{\prime i}= \frac{1+\delta _{a_1}}{1+\delta _{a_1}^i} \mu _{a_2}^{i}, \qquad \mu _{b_2}^{\prime i}= \frac{1+\delta _{b_1}}{1+\delta _{b_1}^i} \mu _{b_2}^{i}. \nonumber\\ \end{eqnarray*}

If it is a signal window, Alice (Bob) randomly chooses sources $a_0,a_z^\prime$ ($b_0,b_z^\prime$) with probabilities 1 − ε_*a*_, ε_*a*_ ( 1 − ε_*b*_, ε_*b*_). Alice (Bob) actually prepares a phase-randomized WCS pulse with intensity $\mu _{a_z}^{\prime i}$ ($\mu _{b_z}^{\prime i}$) if source $a_z^\prime$ ($b_z^\prime$) is chosen, where


(5) \begin{eqnarray*} \mu _{a_z}^{\prime i}= \frac{1+\delta _{a_1}}{1+\delta _{a_1}^i} \mu _{a_z}^{i}, \qquad \mu _{b_z}^{\prime i}= \frac{1+\delta _{b_1}}{1+\delta _{b_1}^i} \mu _{b_z}^{i}. \end{eqnarray*}

Then *they* send out the prepared pulse pair. There are two attenuators between the channel and Alice’s and Bob’s labs, which are controlled by David. To clarify, we define the attenuator on Alice’s side as attenuator A and the attenuator on Bob’s side as attenuator B. In the *i*th time window, David sets the transmittance of attenuator A to $\eta _A^i={(1+\delta _{a_1}^i)}/{(1+\delta _{a_1})}$ and the transmittance of attenuator B to $\eta _B^i={(1+\delta _{b_1}^i)}/{(1+\delta _{b_1})}$. Then we have


(6) \begin{eqnarray*} \mu _{a_1}^i&=&\eta _A^i\mu _{a_1}^U,\quad \mu _{a_2}^{i}=\eta _A^i \mu _{a_2}^{\prime i}, \quad \mu _{a_z}^{i}=\eta _A^i \mu _{a_z}^{\prime i},\nonumber\\ \mu _{b_1}^i&=&\eta _B^i\mu _{b_1}^U,\quad \mu _{b_2}^{i}=\eta _B^i \mu _{b_2}^{\prime i}, \quad \mu _{b_z}^{i}=\eta _B^i \mu _{b_z}^{\prime i}. \nonumber\\ \end{eqnarray*}

**Figure 1.**
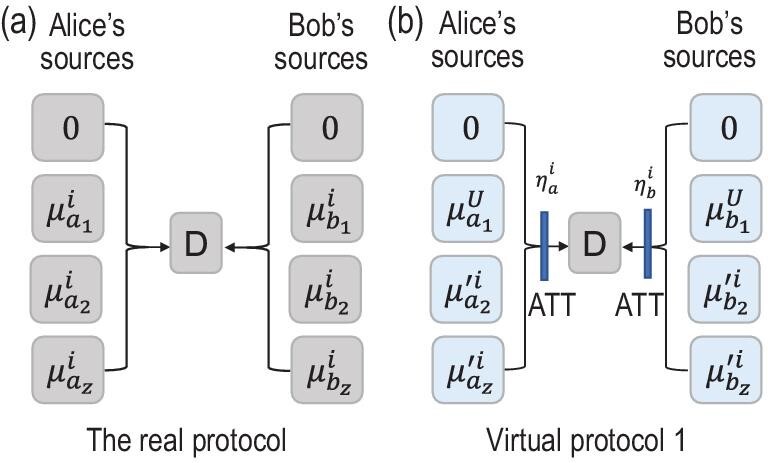
Comparison between the (a) real protocol and (b) virtual protocol 1. Here the boxes labeled ‘D’ represent Charlie’s detectors and ATT represents the attenuator. Although the sources of the real protocol and virtual protocol 1 are different, Eve cannot distinguish the differences due to the proper set attenuators.

Equations (6) mean that the real protocol and virtual protocol 1 are equivalent to Eve who is assumed to control the channel and detectors. Thus, the information leakages in the real protocol and virtual protocol 1 are the same and we can use virtual protocol 1 to estimate the key rate of the real protocol. A simple comparison of the real protocol and virtual protocol 1 is shown in Fig. 1.

Note that in virtual protocol 1 sources $a_1^\prime$ and $b_1^\prime$ are stable, bringing us one step closer to the final goal. However, due to the unstable sources in the *Z* windows, security condition (2) is not always satisfied. Recall that the untagged pulses in the *Z* windows are the single-photon pulses in the $\tilde{Z}$ windows. This means that we should only need to care about the single-photon pulse of sources $a_z^\prime$ and $b_z^\prime$. The density matrix of a phase-randomized WCS pulse with intensity μ is


(7) \begin{eqnarray*} \rho _{\mu }=\sum _{k=0}^{+\infty }\frac{\mu ^ke^{-\mu }}{k!}|k\rangle \langle k|. \end{eqnarray*}

Although source $a_z^\prime$ is unstable, the density matrix of the pulse from this source in the *i*th time window can be expressed in the convex form


(8) \begin{eqnarray*} \rho _{\mu _{a_z}^{\prime i}}=c_{a_z}^1|1\rangle \langle 1|+(1-c_{a_z}^1)\rho _{a_c}^i, \end{eqnarray*}

where


(9) \begin{eqnarray*} \rho _{a_c}^i&=&\frac{1}{1-c_{a_z}^1}\bigg [(\mu _{a_z}^{\prime i}e^{-\mu _{a_z}^{\prime i}}-c_{a_z}^1)|1\rangle\nonumber\\ &&\times \langle 1| + e^{-\mu _{a_z}^{\prime i}}|0\rangle \langle 0|\nonumber \\ && +\sum _{k=2}^{+\infty }\frac{(\mu _{a_z}^{\prime i})^ke^{-\mu _{a_z}^{\prime i}}}{k!}|k\rangle \langle k|\bigg ] \end{eqnarray*}

for $c_{a_z}^1\le \mu _{a_z}^{\prime i}e^{-\mu _{a_z}^{\prime i}}$. And, if $c_{a_z}^1\le \mu _{a_z}^{\prime L}e^{-\mu _{a_z}^{\prime L}}$, the density matrices of all pulses of source $a_z^\prime$ have the similar convex form as shown in (8), where the only differences are $\rho _{a_c}^i$ for different time windows. But, as stated before, we only need to care about the single-photon pulse of source $a_z^\prime$; thus, as long as $c_{a_z}^1\le \mu _{a_z}^{\prime L}e^{-\mu _{a_z}^{\prime L}}$, we can take the unstable source $a_z^\prime$ as the mixture of two sources: one stable source $a_z^{\prime 1}$ with probability $c_{a_z}^1$ and one unstable source $a_z^{\prime c}$ with probability $1-c_{a_z}^1$, where source $a_z^{\prime 1}$ only emits perfect single-photon pulses and source $a_z^{\prime c}$ emits pulses in state $\rho _{a_c}^i$.

For the unstable source $b_z^\prime$, the density matrix of the pulse in the *i*th time window is


(10) \begin{eqnarray*} \rho _{\mu _{b_z}^{\prime i}}=c_{b_z}^1|1\rangle \langle 1|+(1-c_{b_z}^1)\rho _{b_c}^i, \end{eqnarray*}

where


(11) \begin{eqnarray*} \rho _{b_c}^i&=&\frac{1}{1-c_{b_z}^1}\bigg [(\mu _{b_z}^{\prime i}e^{-\mu _{b_z}^{\prime i}}-c_{b_z}^1)|1\rangle\nonumber\\ &&\times \langle 1|+e^{-\mu _{b_z}^{\prime i}}|0\rangle \langle 0|\nonumber \\ && +\,\sum _{k=2}^{+\infty }\frac{(\mu _{b_z}^{\prime i})^ke^{-\mu _{b_z}^{\prime i}}}{k!}|k\rangle \langle k|\bigg ] \end{eqnarray*}

for $c_{b_z}^1\le \mu _{b_z}^{\prime i}e^{-\mu _{b_z}^{\prime i}}$. As long as $c_{b_z}^1\le \mu _{b_z}^{\prime L}e^{-\mu _{b_z}^{\prime L}}$, the unstable source $b_z^\prime$ can be regarded as the mixture of two sources: one stable source $b_z^{\prime 1}$ with probability $c_{b_z}^1$ and one unstable source $b_z^{\prime c}$ with probability $1-c_{b_z}^1$ where source $b_z^{\prime 1}$ only emits perfect single-photon pulses and $b_z^{\prime c}$ emits pulses in state $\rho _{b_c}^i$. With this, we have the following virtual protocol 2.

### Virtual protocol 2

In the *i*th time window, Alice (Bob) randomly decides it is a decoy window or a signal window with probabilities $1-p_{a_z}$ and $p_{a_z}$ ($1-p_{b_z}$ and $p_{b_z}$). If it is a decoy window, Alice (Bob) randomly chooses sources $a_0,a_1^\prime ,a_2^\prime$ ($b_0,b_1^\prime ,b_2^\prime$) with probabilities $p_{a_0}=1-p_{a_1}-p_{a_2},p_{a_1},p_{a_2}$ ($p_{b_0}=1-p_{b_1}-p_{b_2},p_{b_1},p_{b_2}$). If it is a signal window, Alice (Bob) randomly chooses sources $a_0,a_z^{\prime 1}, a_z^{\prime c}$ ($b_0,b_z^{\prime 1}, b_z^{\prime c}$) with probabilities $1-\epsilon _a,\epsilon _a c_{a_z}^1, \epsilon _a(1-c_{a_z}^1)$ [$1-\epsilon _b,\epsilon _b c_{b_z}^1, \epsilon _b(1-c_{b_z}^1)$], where source $a_z^{\prime 1}$ only emits single-photon pulses and source $a_z^{\prime c}$ emits pulses in state $\rho _{a_c}^i$ (source $b_z^{\prime 1}$ only emits single-photon pulses and source $b_z^{\prime c}$ emits pulses in state $\rho _{b_c}^i$), and if


(12) \begin{eqnarray*} \frac{\mu _{a_z}^{\prime L}e^{-\mu _{a_z}^{\prime L}}}{\mu _{b_z}^{\prime L}e^{-\mu _{b_z}^{\prime L}}}\le \frac{\epsilon _b(1-\epsilon _a)\mu _{a_1}^U}{\epsilon _a(1-\epsilon _b)\mu _{b_1}^U}, \end{eqnarray*}

$c_{a_z}^1, c_{b_z}^1$ satisfy


(13) \begin{eqnarray*} c_{a_z}^1=\mu _{a_z}^{\prime L}e^{-\mu _{a_z}^{\prime L}}, \qquad c_{b_z}^1=\frac{\epsilon _a(1-\epsilon _b)\mu _{b_1}^U}{\epsilon _b(1-\epsilon _a)\mu _{a_1}^U}c_{a_z}^1; \nonumber\\ \end{eqnarray*}

otherwise,


(14) \begin{eqnarray*} c_{b_z}^1=\mu _{b_z}^{\prime L}e^{-\mu _{b_z}^{\prime L}}, \qquad c_{a_z}^1=\frac{\epsilon _b(1-\epsilon _a)\mu _{a_1}^U}{\epsilon _a(1-\epsilon _b)\mu _{b_1}^U}c_{b_z}^1. \nonumber\\ \end{eqnarray*}

The following precesses are the same with virtual protocol 1. As discussed above, virtual protocol 2 is equivalent to virtual protocol 1. Eve cannot distinguish the difference between the real protocol, virtual protocol 1 and virtual protocol 2.

We regard all those (single-photon) pulses from sources $\lbrace a_z^{\prime 1}, b_0\rbrace$ and $\lbrace a_0, b_z^{\prime 1}\rbrace$ in *Z* windows as untagged pulses. Clearly, an untagged pulse state here is identical to the single-photon state in *X* windows due to


(15) \begin{eqnarray*} \frac{\mu _{a_1}^U}{\mu _{b_1}^U}=\frac{\epsilon _a(1-\epsilon _b)c_{a_z}^1}{\epsilon _b(1-\epsilon _a)c_{b_z}^1}. \end{eqnarray*}

Furthermore, only sources $a_2^\prime$ and $b_2^\prime$ are unstable in virtual protocol 2. The remaining problems are estimating the lower bound of the number of untagged bits and the upper bound of the phase-flip error rate, both of which can be solved by directly applying the conclusion of [36]. Finally, using the method introduced in [28,29], we can obtain the final key rate. We can also use the AOPP method to improve the key rate [31,32]. The calculation details are shown in Section 1 within the online supplementary material.

### Major results

1. Given the unstable sources as presented in the real protocol, we map them to virtual sources by virtual attenuation and the tagged model, as shown in virtual protocol 2.

2. In virtual protocol 2, sources $a_1^\prime$ and $b_1^\prime$ are stable, with exactly known intensities, and sources $a_z^{\prime 1}$ and $b_z^{\prime 1}$ are perfect single-photon sources, while other non-vacuum sources are unstable, with known bounds.

3. Regard the observed values in the real protocol to be those of the virtual protocol, and use decoy-state analysis to verify the values $n_1^L$ and $e_1^{ph,U}$, which are the lower bound value of the number of untagged bits in *Z* windows and the upper bound value of the phase-flip error rate.

4. Calculate the key rate for the real protocol using the formula


(16) \begin{eqnarray*} R&=&\frac{1}{N}\bigg \lbrace n_1^L[1-H(e_1^{ph,U})]-fn_tH(E)\nonumber \\ && -\, \log _2 \frac{2}{\varepsilon _{\scriptsize\rm{\it cor}}}-2\log _2\frac{1}{\sqrt{2}\varepsilon _{PA}\hat{\varepsilon }}\bigg \rbrace , \end{eqnarray*}

where *N* represents the total number of pulse pairs sent by Alice and Bob, *f* represents the error correction inefficiency, *n_t_* represents the number of effective events in the *Z* windows and *E* represents the error rate of the raw keys in the *Z* windows. As shown in [19,29], the tailing term of $-\log _2 ({2}/{\varepsilon _{cor}})-2\log _2({1}/{\sqrt{2}\varepsilon _{PA}\hat{\varepsilon }})$ is the additional cost for security with finite size. The calculation details are shown in Section 1 within the online supplementary material. As detailed in the online supplementary material, our method does not presume the source errors to be random. Our protocol assumes that Eve can know or even determine the error values of all those candidate states in advance, so they surely are not limited to being random errors only. For security, the decoy-state method requests that Eve has no information in advance about the secret state choice of Alice and Bob, i.e. choosing the pulse of which source to send out by Alice and Bob for each time window. To keep this condition, our method requests that the values of intensity errors that can be known to Eve in advance do not carry any private information about the state choice of Alice and Bob. With this presumption being respected, our method allows whatever dependence of intensity errors from different time windows.

5. The AOPP method is directly applicable here. Suppose that Bob generates the active odd-parity random pairing and then informs Alice of the positions of two bits in raw keys for each pair. Following the parity check, all pairs with odd-parity values at Alice’s side survive, and one bit from each survived pair is chosen at random for final key distillation. That is, in the AOPP, a pair with two untagged bits will always contribute an untagged bit to the final key distillation. Those pairs with even-parity values on Alice’s side will be completely discarded. We have the following final key length formula after AOPP:


(17) \begin{eqnarray*} R^\prime &=&\frac{1}{N}\bigg \lbrace n_1^{\prime L}[1-H(e_{1}^{\prime ph,U})]-fn_t^\prime H(E^\prime ) \nonumber \\ && -\, 2\log _2{\frac{2}{\varepsilon _{\scriptsize\rm{\it cor}}}}-4\log _2{\frac{1}{\sqrt{2}\varepsilon _{PA} \hat{\varepsilon }}}\bigg \rbrace . \end{eqnarray*}

Here $n_1^{\prime L}$ is the lower bound of untagged bits after AOPP, $e_{1}^{\prime ph,U}$ is the upper bound of the phase-flip error rate after AOPP, $n_t^\prime$ is the number of survived bits after AOPP and *E*′ is the bit-flip error rate of the survived bits after AOPP. Details are shown in Section 1 within the online supplementary material.

## NUMERICAL SIMULATION

We use the linear model to simulate the observed values [28], and Charlie’s detectors are assumed to be identical, that is, they have the same detection efficiency and dark counting rate. For simplicity, we assume that the maximum derivations of all sources are the same, i.e. $\delta _{a_l} = \delta _{b_r} = \delta$ for *l*, *r* = 1, 2, *z*. The experimental parameters are listed in Table 1.

**Table 1.** List of experimental parameters used in numerical simulations. Here *p_d_* is the dark counting rate per pulse of Charlie’s detectors, *e_d_* is the misalignment-error probability, η_*d*_ is the detection efficiency of Charlie’s detectors, *f* is the error correction inefficiency, α_*f*_ is the fiber loss coefficient (*dB*/*km*), ξ is the failure probability while using Chernoff’s bound [39] and *N* is the number of total pulse pairs sent out in the protocol.


*p_d_*	*e_d_*	η_*d*_	*f*	α_*f*_	ξ	*N*

1.0 × 10^−8^	$4\%$	$60.0\%$	1.1	0.2	1.0 × 10^−10^	1.0 × 10^13^

**Figure 2.**
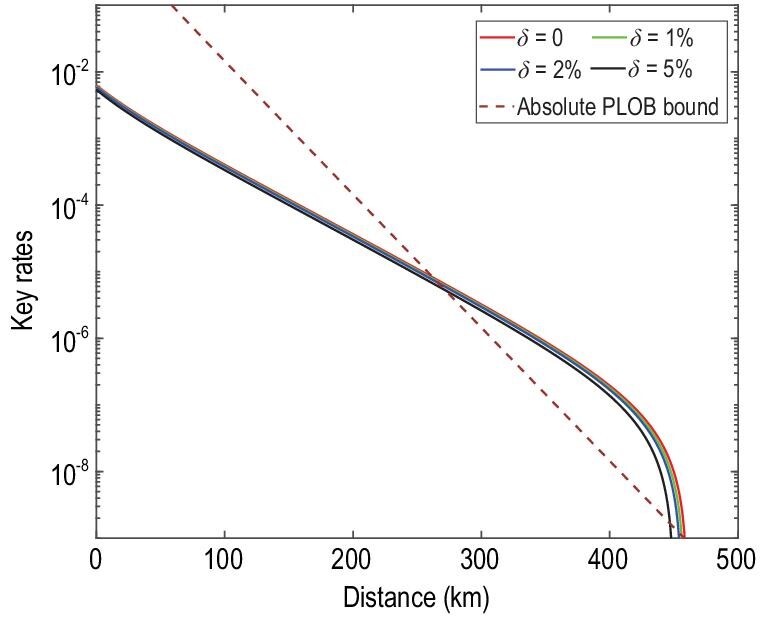
The key rates of the original SNS protocol under different degrees of intensity fluctuation. The absolute PLOB bound is the PLOB bound with $100\%$ detection efficiency detectors. Here we assume the symmetric channel, i.e. the distance between Alice and Charlie, *L_AC_*, equals the distance between Bob and Charlie, *L_BC_*. The experimental parameters are listed in Table 1.

**Figure 3.**
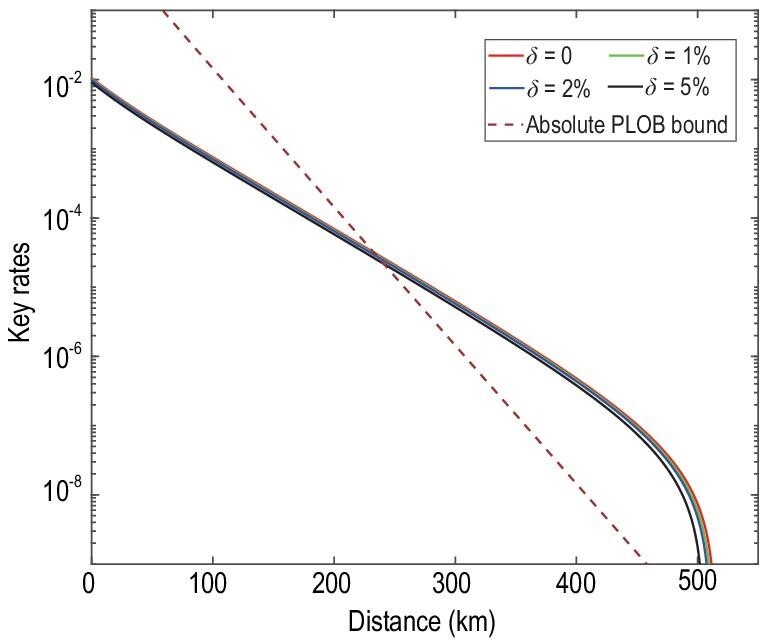
The key rates of the SNS protocol with AOPP under different degrees of intensity fluctuation. The absolute PLOB bound is the PLOB bound with $100\%$ detection efficiency detectors. Here we assume the symmetric channel, i.e. the distance between Alice and Charlie, *L_AC_*, equals the distance between Bob and Charlie, *L_BC_*. The experimental parameters are listed in Table 1.

**Figure 4.**
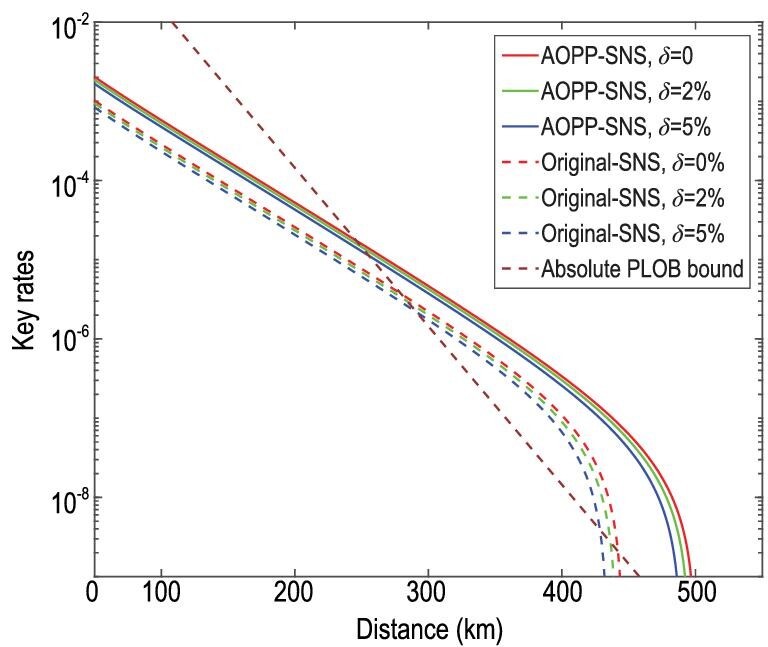
Comparison between the key rates of the original SNS protocol and the SNS protocol with AOPP method under different degrees of intensity fluctuation. The absolute PLOB bound is the PLOB bound with $100\%$ detection efficiency detectors. Here we assume the asymmetric channel, where the distance between Alice and Charlie, *L_AC_*, and the distance between Bob and Charlie, *L_BC_*, satisfy *L_AC_* − *L_BC_* = 50 km. The experimental parameters are listed in Table 1.

Figures 2 and 3 are the key rates of the original SNS protocol and the SNS protocol with the AOPP method under different degrees of intensity fluctuation. We assume a symmetric channel, which means that the distance between Alice and Charlie, *L_AC_*, equals the distance between Bob and Charlie, *L_BC_*. Figures 2 and 3 show that the key rates and farthest distance decrease slightly as the intensity fluctuation range increases. Figure 4 shows the comparison between the key rates of the original SNS protocol and the SNS protocol with the AOPP method under different degrees of intensity fluctuation, where we set *L_AC_* − *L_BC_* = 50 km. The key rates at a distance of 350 km are shown in Table 3 below, where the experimental parameters are the same as those of Fig. 4. In Figs 2–4, the absolute PLOB bound is for the case with perfect detection efficiency. The results in those figures show that the key rates of our method can still exceed the absolute PLOB bound with $5\%$ intensity fluctuation. As the intensity fluctuation increases, so does the distance corresponding to the first intersection between the absolute PLOB bound and the key rate curve, implying that it is more difficult to exceed the absolute PLOB bound with a larger intensity fluctuation; this trend is more apparent in Fig. 4. The key rates and distances corresponding to the first intersections in Figs 2–4 are shown in Table 2.

**Table 2.** The key rates and distances corresponding to the first intersections between the absolute PLOB bound and the key rate curves in Figs 2–4. Here the results of the original method with symmetric channel are the intersections in Fig. 2; the results of the AOPP method with symmetric channel are the intersections in Fig. 3; the results of the AOPP and original methods with asymmetric channel are the intersections in Fig. 4.

	
	δ					
	$0\%$	$2\%$	$5\%$	$0\%$	$2\%$	$5\%$
	
Channel	Symmetric	Symmetric	Symmetric	Symmetric	Symmetric	Symmetric
Method	AOPP	AOPP	AOPP	Original	Original	Original
Distance	233	237	241	263	267	272
Key rate	3.16 × 10^−5^	2.67 × 10^−5^	2.18 × 10^−5^	8.00 × 10^−6^	6.66 × 10^−6^	5.24 × 10^−6^
Channel	Asymmetric	Asymmetric	Asymmetric	Asymmetric	Asymmetric	Asymmetric
Method	AOPP	AOPP	AOPP	Original	Original	Original
Distance	246	250	256	280	284	291
Key rate	1.73 × 10^−5^	1.44 × 10^−5^	1.11 × 10^−5^	3.66 × 10^−6^	3.00 × 10^−6^	2.18 × 10^−6^

**Table 3.** The key rates of the original SNS protocol and the AOPP method under different degrees of intensity fluctuation. The distance between Alice and Bob is 350 km, and the experimental parameters are the same as those of Fig. 4. In our simulation, the fluctuation parameter δ is between −δ and δ. For example, $\delta =10\%$ means a fluctuating range of $20\%$, from $-10\%$ to $+10\%$.

	
	δ			
	$0\%$	$2\%$	$5\%$	$10\%$
	
Original SNS	5.8 × 10^−7^	5.15 × 10^−7^	4.35 × 10^−7^	3.26 × 10^−7^
AOPP SNS	1.33 × 10^−6^	1.20 × 10^−6^	1.05 × 10^−6^	8.46 × 10^−7^

We use a more efficient method to calculate the key rates of the AOPP SNS in Table 3, by scanning the expected value of the counting rate of vacuum sources 〈*S*_00_〉 in its range, which is


(18) \begin{eqnarray*} R^{\prime \prime }=\min _{\langle S_{00}\rangle }R^{\prime }(\langle S_{00}\rangle ), \end{eqnarray*}

and this can improve the non-asymptotic key rate a little bit. The results in Table 3 show that the key rates of AOPP with $5\%$ intensity fluctuation are still higher than those of the original SNS protocol without intensity fluctuation. From Table 3 we can see that every $1\%$ intensity fluctuation causes a $4\%$ drop in the key rate, which shows that the SNS protocol is robust against source errors.

The AOPP method was used in the SNS experiment in a 511-km field fiber to achieve a higher key rate [8]. We calculate the key rate of this experiment if there are source errors using the experimentally observed values shown in [8], and the results show that the key rate is still positive even if δ is as large as $9.5\%$.

In the security proof and numerical simulation above, we assume that the intensities of the pulses are in a certain interval. But a more practical case is that very few pulses can exceed the bound values and we can only determine at most the number of pulses outside the interval with a small failure probability. We show how to calculate the key rate under this case in Section 4 within the online supplementary material.

## CONCLUSION

In this study, we propose a strict method for calculating the key rate of the SNS protocol with source errors, by establishing the equivalent protocols through virtual attenuation and the tagged model. We finally obtain the key rate formulas under the premise of ensuring the protocol’s security. Our method can be combined with the AOPP method to further improve the key rate. The numerical results show that every $1\%$ intensity fluctuation causes a $4\%$ drop in the key rate; additionally, the SNS protocol’s farthest distance slightly decreases as the intensity fluctuation range increases, indicating that the SNS protocol is robust against source errors. Our method can be directly applied to the three-intensity SNS protocol with source error where ‘three intensity’ refers to $\mu _{a_2} = \mu _{a_z}, \mu _{b_2} = \mu _{b_z}{.}$

Since our method, on the one hand, allows Eve to know the error values of candidate pulses in advance and, on the other hand, requests no information leakage of the state choice, the errors must be independent of the state choice in applying our method [40]. With this condition being respected, our method applies to errors inside the bound values with whatever patterns or correlations of intensities among different time windows, as shown in the online supplementary material. Our method does not work with crossing correlations between intensity errors and the state choice at different time windows, say, setting-choice-dependent errors [41–43], because such types of crossing correlations obviously lead to the state-choice information leakage to Eve if she knows the error values in advance. It is an interesting problem for future study on the setting-choice-dependent errors [41–43] with TF-QKD protocols.

## Supplementary Material

nwac186_Supplemental_File
