# Supplementary material for: Robust twin-field quantum key distribution through sending or not sending
Source: Natl Sci Rev. 2022 Sep 19;10(4):nwac186. doi: 10.1093/nsr/nwac186 (PMC10115169; doi:10.1093/nsr/nwac186)
Supplement: nwac186_Supplemental_File [file nwac186_supplemental_file.zip › main-red.pdf]

RESEARCH ARTICLE

National Science Review
XX: main-red, Year
doi: https://doi.org/10.1093/nsr/XXXX

PHYSICS

Robust twin-field quantum key distribution through sending-or-not-sending

Cong Jiang[1,2], Zong-Wen Yu[3], Xiao-Long Hu[4] and Xiang-Bin Wang[1,2,5,6,7,*]

[1] Jinan Institute of Quantum Technology, Jinan, Shandong 250101, P. R. China; [2]State Key Laboratory of Low Dimensional Quantum Physics, Department of Physics, Tsinghua University, Beijing 100084, P. R. China; [3]Data Communication Science and Technology Research Institute, Beijing 100191, P. R. China; [4]School of Physics, State Key Laboratory of Optoelectronic Materials and Technologies, Sun Yat-sen University, Guangzhou 510275, China; [5]Shanghai Branch, CAS Center for Excellence and Synergetic Innovation Center in Quantum Information and Quantum Physics, University of Science and Technology of China, Shanghai 201315, P. R. China; [6]Shenzhen Institute for Quantum Science and Engineering, and Physics Department, Southern University of Science and Technology, Shenzhen 518055, China; [7]Frontier Science Center for Quantum Information, Beijing, China

*Corresponding authors. Email: xb-wang@mail.tsinghua.edu.cn.

Received: XX XX Year; **Revised:** XX XX Year; **Accepted:** XX XX Year

ABSTRACT

The sending-or-not-sending (SNS) protocol is one of the most major variants of the twin-field (TF) quantum key distribution (QKD) protocol and has been realized in 511 km field fiber, the farthest field experiments to date. In practice, however, all decoy-state methods have unavoidable source errors, and the source errors may be non-random. In practice, the non-random source errors compromise the security condition of the existing TF-QKD protocols. In this study, we present a general approach for efficiently calculating the SNS protocol's secure key rate with source errors, by establishing the equivalent protocols through virtual attenuation and tagged model. This makes the first result for TF-QKD in practice where source intensity cannot be controlled exactly. Our method can be combined with the two-way classical communication method such as active odd-parity pairing to further improve the key rate. The numerical results show that if the intensity error is not too large, say a few percent, the key rate and secure distance only decrease marginally. The key rate of the recent SNS experiment in 511 km field fiber is still positive using our method presented here, even if there is 9.5% intensity fluctuation, i.e., a fluctuating range of 19%, from –9.5% to +9.5%. This shows that the SNS protocol is robust against source errors.

Keywords: Quantum information, Quantum key distribution, Sending-or-not-sending, Twin-field, Source error

Introduction

Since the proposal of the twin-field (TF) quantum key distribution (QKD) [1], the longest distance record of QKD has been constantly and rapidly refreshed in recent years [2–9]. The upper bounds of the key rate of the previous protocols, such as the BB84 protocol [10–15] and the measurement-device-independent(MDI) QKD protocol [16–22] are limited to the linear scale of channel transmittance, also known as the PLOB bound [23]. Based on the single-photon interference, the TF-QKD can raise the key rate from linear scale to square root scale of channel transmittance and break the PLOB bound.

As one of the most important variants of TF QKD [24–27], the sending-or-not-sending (SNS) protocol [24] has been extensively studied in theories [28–32] and experiments [2,6–9,33]. Specifically, the 511-km field experiment [8] was done by applying the SNS protocol with actively odd-parity pairing (AOPP) method, and the 605-km laboratory experiment [9] was done by applying the SNS protocol with standard two-way classical communication (TWCC) method. The 511-km experiment is the farthest field experiment to date.

The TF-QKD has typically used with the weak coherent state (WCS) sources. The decoy-state method must be used to ensure the security of TF-QKD with WCS sources. The intensities of the WCS sources are always assumed to be stable in the entire protocol, which is not the fact in experiments where source errors are unavoidable. The source errors, in particular, are not always random. For example, the intensities of the sources can slowly shift over time and are affected by temperature changes in the environment.

Although there are results for decoy-state analysis with source errors for the BB84 and the MDIQKD protocol [34,35], they do not solve the problem for the SNS protocol where encoding is done by the vacuum and single-photon states themselves. To calculate the key rate, we need a way to estimate the single-photon phase-flip error rate. It is not a trivial task because the single

© The Author(s) Year. Published by Oxford University Press on behalf of China Science Publishing & Media Ltd. This is an Open Access article distributed under the terms of the Creative Commons Attribution License (http://creativecommons.org/licenses/by/4.0/), which permits unrestricted reuse, distribution, and reproduction in any medium, provided the original work is properly cited.

photon state in X basis is now different from that in Z basis. We shall solve this problem by virtual attenuation and then apply the existing theory for decoy-state analysis with intensity fluctuation.

The work is organized as follows: first, we introduce the real and virtual protocols and demonstrate their equivalence. This shows how to efficiently calculate the secure key rate in a practical application where intensities cannot be controlled exactly. Then we present some numerical results that compare the key rates of the SNS protocol with different degrees of source errors. The article is ended with conclusion remarks.

The security of SNS protocol with source errors

For ease of presentation, here we introduce the SNS protocol through the model of using different sources. The scientific content is the same with the earlier literatures.

We take the 4-intensity SNS protocol as an example to show the security proof. There are four sources with different intensities on Alice's and Bob's sides. We denote Alice's sources by a_0, a_1, a_2 and a_z, and Bob's sources by b_0, b_1, b_2 and b_z, where a_0 and b_0 are the vacuum sources. WCS sources are typically used to perform the SNS protocol. If the sources are stable, the intensities of the sources a_l and b_r are μ_{a_l} and μ_{b_r}, respectively, for $l, r = 0, 1, 2, z$ where $\mu_{a_0} = \mu_{b_0} = 0$. However, in practice, the sources are always unstable and the intensities are different in different time windows. The intensities of the sources in the i-th time window are denoted as $\mu^i_{a_l}$ and $\mu^i_{b_r}$ respectively, where

$$\mu^i_{a_l} = (1 + \delta^i_{a_l})\mu_{a_l}, \quad \mu^i_{b_r} = (1 + \delta^i_{b_r})\mu_{b_r} \quad (1)$$

and $|\delta^i_{a_l}| \le \delta_{a_l}, |\delta^i_{b_r}| \le \delta_{b_r}$. We assume δ_{a_l} and δ_{b_r} are known values in the protocol. The lower and upper bounds of a physical quantity are represented by superscripts L and U, respectively. For instance, $\mu^U_{a_1}$ is the upper bound of the intensity of the source a_1, and we have $\mu^L_{a_1} = (1 + \delta_{a_1})\mu_{a_1}$.

The real protocol

Alice (Bob) randomly decides whether the i-th time window is a decoy window or a signal window with probabilities $1 - p_{a_z}$ and p_{a_z} ($1 - p_{b_z}$ and p_{b_z}). If it is a decoy window, Alice (Bob) randomly chooses the sources a_0, a_1, a_2 (b_0, b_1, b_2) with probabilities $p_{a_0} = 1 - p_{a_1} - p_{a_2}, p_{a_1}, p_{a_2}$ ($p_{b_0} = 1 - p_{b_1} - p_{b_2}, p_{b_1}, p_{b_2}$). Since the sources are unstable, Alice (Bob) actually prepares a WCS pulse in state $\left|e^{\imath\theta^i_{a_l}}\sqrt{\mu^i_{a_l}}\right\rangle$ ($\left|e^{\imath\theta^i_{b_r}}\sqrt{\mu^i_{b_r}}\right\rangle$) if the source a_l (b_r) is chosen for $l, r = 1, 2$, where θ_{a_l} and θ_{b_r} are random in $[0, 2\pi)$. The imaginary unit is represented here by the symbol $\imath$. If it is a signal window, Alice (Bob) randomly chooses the sources a_0, a_z (b_0, b_z) with probabilities $1 - \epsilon_a, \epsilon_a$ ($1 - \epsilon_b, \epsilon_b$). Alice (Bob) actually prepares a phase-randomized WCS pulse with intensity $\mu^i_{a_z}$ ($\mu^i_{b_z}$) if the source a_z (b_z) is chosen. Clearly, if Alice (Bob) commits to a signal window at time i, Alice's (Bob's) choice of source a_z (b_z) indicates a decision of *sending* whereas the choice of source a_0 (b_0) is a decision of *not-sending* in SNS protocol.

They (Alice and Bob) send the prepared pulse pair to Charlie, who is assumed to perform interferometric measurements on the received pulse pair. Then Charlie announces the measurement results to *them*. If only one detector clicks, *they* would take it as a *one-detector heralded event*. After *they* repeat the above process for N times and Charlie announces all measurement results, *they* acquire a series of data. Then *they* perform the data post processing, including post selection of events in X windows and final key distillation.

They first announce the type of each time window they decide, i.e., whether it is a decoy window or a signal window. For a time window that both *them* decide a signal window, it is a Z window. The one-detector heralded events in Z windows are effective events, and the corresponding bits of those effective events formed the n_t-bit raw key strings, which are used to extract the final keys. We also define the $\tilde{Z}$ window as a Z window when sources $\{a_0, b_z\}$ or $\{a_z, b_0\}$ are used, i.e., a Z window when one side decides sending and the other side decides not-sending. The untagged pulses in the Z windows are Alice decides not sending and Bob actually sends out a single-photon pulse or Bob decides not sending and Alice actually sends out a single-photon pulse, i.e., the single-photon pulses in the $\tilde{Z}$ windows. The untagged bits are the corresponding bits of those untagged pulses that cause effective events. Except the Z windows, *they* announce the intensities of the pulses in each window, and for the time windows that both *them* decide sending out a pulse of source a_1 and b_1 respectively, *they* also announce the phases of the pulse pairs. For a time window that both *them* decide sending out a pulse of source a_1 and b_1 respectively, and their phases satisfy the post selection criteria [24], it is an X window. The one-detector her-

alded events in X windows are effective events. The effective events in X windows are used to estimate the phase-flip error rate.

According to Ref. [30], if the sources are stable, and source a_z (b_z) always emits a state of $\rho_{a_z} = \sum_{k=0} q_A^k |k\rangle\langle k|$, ($\rho_{b_z} = \sum_{k=0} q_B^k |k\rangle\langle k|$), we can use the following constraint for the source for security,

$$\frac{\mu_{a_1}}{\mu_{b_1}} = \frac{\epsilon_a(1-\epsilon_b)q_A^1}{\epsilon_b(1-\epsilon_a)q_B^1}, \tag{2}$$

Here $|k\rangle\langle k|$ represents for a k-photon Fock state. In particular, when using a phase randomized WCS source with intensity μ_{a_z} (μ_{b_z}) for source a_z (b_z), q_A^1, q_B^1 in Eq. (2) can be replaced by $\mu_{a_z} e^{-\mu_{a_z}}, \mu_{b_z} e^{-\mu_{b_z}}$. Note that if the constraint above is respected, the density matrix of single-photon state in $\tilde{Z}$ windows is identical to that of X basis and hence we can faithfully verify the phase-flip error rate of untagged bits by observing events in X windows.

Given stable sources with the source parameters known, the SNS protocol is still secure and efficient even if Eq. (2) does not hold. Most straightforwardly, we can post select the sending probabilities ϵ_a, ϵ_b by randomly deleting some of the time windows [6], i.e., to make Eq. (2) hold by post deletion. (Post deleting a time window means disregarding the event happening at that time window.) Although the amount of data is reduced by the post deletion, the key rate will essentially be unchanged if the original bias between the two sides of Eq. (2) is not too large, because, after post deletion, the error rate of raw keys is also decreased.

The more practical situation is that we do not know the exact intensities of the sources of the Z windows but we know the bound values of intensities for each source of Z windows. In such a case, we can use the tagged model: replace the intensities of Eq. (2) by a lower bound value and make Eq. (2) hold.

Most generally, the source intensities of X windows are also unstable. Say, in practice, none of the sources are stable, and the intensity errors may be different from time to time and not necessarily random. In such a case, Eve can treat different time windows differently, thus we can not treat the X windows as a whole, but have to deal with each time window separately. The intensities of sources a_1, b_1 in different time windows are different, which means in general

$$\frac{\mu_{a_1}^i}{\mu_{b_1}^i} \neq \frac{\mu_{a_1}^j}{\mu_{b_1}^j}, \tag{3}$$

if $i \neq j$. When Eq. (2) holds, the bit-flip error rate of X windows can be used to estimate the phase-flip error rate of the untagged bits in Z windows. But due to the source errors, Eq. (2) can not be held for all X windows, which results that we can not estimate the phase-flip error rate of the untagged bits according to the data in X windows. The situation is different for the decoy-state BB84 protocol with source errors [34], or the decoy-state MDIQKD [16,22] where the source errors do not change the encoding states in X windows or Z windows, it affects the decoy-state analysis only [34,35]. In this work, we solve this problem based on SNS protocol and makes the TF-QKD robust with imperfect real set-ups.

Main idea: Quantum security from virtual attenuation

The state preparation stage of the real protocol can be regarded as this: At every time window i, Alice (Bob) has 4 candidate states emitted from the 4 sources a_0, a_1, a_2, a_z (b_0, b_1, b_2, b_z), respectively. In particular, the intensities of the coherent states emitted by source a_l, b_r are $\mu_{a_l}^i, \mu_{b_r}^i$ for $l, r = 1, 2, z$, respectively. Sources a_0, b_0 emit vacuum only. Alice (Bob) will choose one state from the 4 candidate states for the time window i, by the probabilities stated earlier in the real protocol.

Our first major idea is mapping by (virtual) attenuation [36]. We regard the candidate states above as the attenuation outcome from virtual sources a_0, a_1', a_2', a_z' (b_0, b_1', b_2', b_z') and the intensity of the WCS pulse from virtual source a_1' (b_1') is fixed to $\mu_{a_1}^U$ ($\mu_{b_1}^U$) exactly at any time window i. This naturally means that the transmittance $\eta_A^i = \frac{1+\delta_{a_1}^i}{1+\delta_{a_1}}$ ($\eta_B^i = \frac{1+\delta_{b_1}^i}{1+\delta_{b_1}}$) in the virtual attenuation of the i-th time window, and hence the intensities of the WCS pulses from virtual sources a_2', a_z' (b_2', b_z') are $\mu_{a_2}'^i = \mu_{a_2}^i/\eta_A^i, \mu_{a_z}'^i = \mu_{a_z}^i/\eta_A^i$, ($\mu_{b_2}'^i = \mu_{b_2}^i/\eta_B^i, \mu_{b_z}'^i = \mu_{b_z}^i/\eta_B^i$), respectively. For simplicity, we shall just call virtual protocol (real protocol) for the SNS protocol using virtual (real) sources above. Surely, the real protocol above is secure provided that the virtual protocol above is secure. This holds even though Eve knew the exact values of intensity fluctuation of each source in every time window i. Or equivalently, even though Eve knew the exact values of η_a^i, η_b^i. If Eve can use scheme $\mathcal{G}$ to attack the real protocol, Eve can also attack the virtual protocol by first taking attenuation $\{\eta_A^i, \eta_B^i\}$ to pulses in the virtual protocol and then use attacking scheme $\mathcal{G}$. Surely, we

can obtain the secure key rate for the real protocol above by calculating the secure key rate of the virtual protocol, because the two protocols cause no difference to outside lab.

Our second major idea is to treat the intensity fluctuation for states of the Z windows by the tagged model. Consider the key rate calculation for our virtual protocol above. In that protocol, at time window i, the intensity of the pulse from virtual source a_z' (b_z') is μ'_{a_z} (μ'_{b_z}). This in general does not respect Eq. (2). However, in $\tilde{Z}$ windows, there does exist a subset of single-photon pulses which are indistinguishable from the single-photons in X windows, and there does exist an efficient method to verify the lower bound of the size of this subset. Thus the key rate can be effectively calculated.

The third major idea here is to verify the lower bound values of the untagged bits in Z windows and their corresponding upper bound value of phase flip error rate, by applying the existing decoy-state method with source errors [34]. Note that in the virtual protocol above, the intensities of the stronger decoy pulses are not stable. According to the existing theory, we only need to use its lower bound values of the photon number distribution coefficients to obtain the worst-case result. With these, the final key rate can be calculated. Furthermore, we can apply the TWCC method such as AOPP to improve the key rate [31,32].

The virtual protocol 1

In the $i-$th time window of virtual protocol 1, the pulse emitted by sources a_1, a_2, a_z (b_1, b_2, b_z) are different from those of the real protocol. To avoid confusion, we use notations a_0, a_1', a_2', a_z' (b_0, b_1', b_2', b_z') for Alice's (Bob's) sources in virtual protocol 1.

In the i-th time window, Alice (Bob) randomly decides it is a decoy window or a signal window with probabilities $1 - p_{a_z}$ and p_{a_z} ($1 - p_{b_z}$ and p_{b_z}). If it is a decoy window, Alice (Bob) randomly chooses the sources a_0, a_1', a_2' (b_0, b_1', b_2') with probabilities $p_{a_0} = 1 - p_{a_1} - p_{a_2}, p_{a_1}, p_{a_2}$ ($p_{b_0} = 1 - p_{b_1} - p_{b_2}, p_{b_1}, p_{b_2}$). If the source a_1' (b_1') is chosen, Alice (Bob) actually prepares a WCS pulse in state $\left|e^{\imath\theta^i_{a_1}}\sqrt{\mu^U_{a_1}}\right\rangle$ ($\left|e^{\imath\theta^i_{b_1}}\sqrt{\mu^U_{b_1}}\right\rangle$), where $\mu^U_{a_1} = (1+\delta_{a_1})\mu_{a_1}$ and $\mu^U_{b_1} = (1+\delta_{b_1})\mu_{b_1}$. If the source a_2' (b_2') is chosen, Alice (Bob) actually prepares a WCS pulse

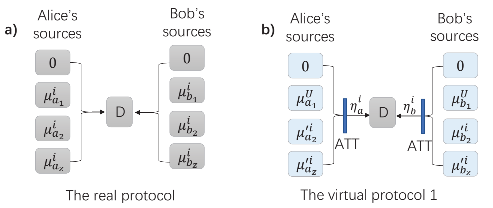

Figure 1. The comparison of the real protocol and the virtual protocol 1. Here the box 'D' represents Charlie's detectors and the 'ATT' represents the attenuator. Although the sources of the real protocol and the virtual protocol 1 are different, Eve cannot distinguish the differences due to the proper set attenuators.

in state $\left|e^{\imath\theta^i_{a_2}}\sqrt{\mu'^i_{a_2}}\right\rangle$ ($\left|e^{\imath\theta^i_{b_2}}\sqrt{\mu'^i_{b_2}}\right\rangle$), where

$$\mu'^i_{a_2} = \frac{1+\delta_{a_1}}{1+\delta^i_{a_1}}\mu^i_{a_2}, \quad \mu'^i_{b_2} = \frac{1+\delta_{b_1}}{1+\delta^i_{b_1}}\mu^i_{b_2}. \tag{4}$$

If it is a signal window, Alice (Bob) randomly chooses the sources a_0, a_z' (b_0, b_z') with probabilities $1 - \epsilon_a, \epsilon_a$ ($1 - \epsilon_b, \epsilon_b$). Alice (Bob) actually prepares a phase-randomized WCS pulse with intensity $\mu'^i_{a_z}$ ($\mu'^i_{b_z}$) if the source a_z' (b_z') is chosen, where

$$\mu'^i_{a_z} = \frac{1+\delta_{a_1}}{1+\delta^i_{a_1}}\mu^i_{a_z}, \quad \mu'^i_{b_z} = \frac{1+\delta_{b_1}}{1+\delta^i_{b_1}}\mu^i_{b_z}. \tag{5}$$

Then *they* send out the prepared pulse pair. There are two attenuators between the channel and Alice's and Bob's labs, which are controlled by David. To clarify, we define the attenuator on Alice's side as attenuator A and the attenuator on Bob's side as attenuator B. In the $i-$th time window, David sets the transmittance of the attenuator A to $\eta^i_A = \frac{1+\delta^i_{a_1}}{1+\delta_{a_1}}$, and the transmittance of the attenuator B to $\eta^i_B = \frac{1+\delta^i_{b_1}}{1+\delta_{b_1}}$. Then we have

$$\mu^i_{a_1} = \eta^i_A\mu^U_{a_1}, \quad \mu^i_{a_2} = \eta^i_A\mu'^i_{a_2}, \quad \mu^i_{a_z} = \eta^i_A\mu'^i_{a_z},$$
$$\mu^i_{b_1} = \eta^i_B\mu^U_{b_1}, \quad \mu^i_{b_2} = \eta^i_B\mu'^i_{b_2}, \quad \mu^i_{b_z} = \eta^i_B\mu'^i_{b_z}. \tag{6}$$

Eq. (6) means the real protocol and virtual protocol 1 are equivalent to Eve who is assumed to control the channel and detectors. Thus the information leakages in the real protocol and virtual protocol 1 are the same and we can use virtual protocol 1 to estimate the key rate of the real protocol. A simple comparison of the real protocol and virtual protocol 1 is shown in Figure. 1.

Note in virtual protocol 1, the sources a_1' and b_1' are stable, bringing us one step closer to the final goal. However, due to the unstable sources in the Z windows, the security condition Eq. (2)

is not always satisfied. Recall that the untagged pulses in the Z windows are the single-photon pulses in the $\tilde{Z}$ windows. This means that we should only need to care about the single-photon pulse of the sources a'_z and b'_z. The density matrix of a phase-randomized WCS pulse with intensity μ is

$$\rho_\mu = \sum_{k=0}^{+\infty} \frac{\mu^k e^{-\mu}}{k!} |k\rangle\langle k|. \tag{7}$$

Although the source a'_z is unstable, the density matrix of the pulse from this source in the i-th time window can be expressed in the following convex form

$$\rho_{\mu'^i_{a_z}} = c^1_{a_z}|1\rangle\langle 1| + (1 - c^1_{a_z})\rho^i_{a_c}, \tag{8}$$

where

$$\begin{aligned}\rho^i_{a_c} = & \frac{1}{1 - c^1_{a_z}}[(\mu'^i_{a_z} e^{-\mu'^i_{a_z}} - c^1_{a_z})|1\rangle\langle 1| + \\ & e^{-\mu'^i_{a_z}}|0\rangle\langle 0| + \sum_{k=2}^{+\infty} \frac{(\mu'^i_{a_z})^k e^{-\mu'^i_{a_z}}}{k!}|k\rangle\langle k|],\end{aligned} \tag{9}$$

for $c^1_{a_z} \leq \mu'^i_{a_z} e^{-\mu'^i_{a_z}}$. And if $c^1_{a_z} \leq \mu'^L_{a_z} e^{-\mu'^L_{a_z}}$, the density matrices of all pulses of source a'_z have the similar convex form as shown in Eq. (8), where the only differences are $\rho^i_{a_c}$ for different time windows. But as stated before, we only need to care about the single-photon pulse of the source a'_z, thus as long as $c^1_{a_z} \leq \mu'^L_{a_z} e^{-\mu'^L_{a_z}}$, we can take the unstable source a'_z as the mixture of two sources: one stable source a'^1_z with probability $c^1_{a_z}$ and one unstable source a'^c_z with probability $1 - c^1_{a_z}$, where the source a'^1_z only emits perfect single-photon pulses and the source a'^c_z emits pulses in state $\rho^i_{a_c}$.

For the unstable source b'_z, the density matrix of the pulse in $i-$th time window is

$$\rho_{\mu'^i_{b_z}} = c^1_{b_z}|1\rangle\langle 1| + (1 - c^1_{b_z})\rho^i_{b_c}, \tag{10}$$

where

$$\begin{aligned}\rho^i_{b_c} = & \frac{1}{1 - c^1_{b_z}}[(\mu'^i_{b_z} e^{-\mu'^i_{b_z}} - c^1_{b_z})|1\rangle\langle 1| + \\ & e^{-\mu'^i_{b_z}}|0\rangle\langle 0| + \sum_{k=2}^{+\infty} \frac{(\mu'^i_{b_z})^k e^{-\mu'^i_{b_z}}}{k!}|k\rangle\langle k|],\end{aligned} \tag{11}$$

for $c^1_{b_z} \leq \mu'^i_{b_z} e^{-\mu'^i_{b_z}}$. As long as $c^1_{b_z} \leq \mu'^L_{b_z} e^{-\mu'^L_{b_z}}$, the unstable source b'_z can be regarded as the mixture of two sources: one stable source b'^1_z with probability $c^1_{b_z}$ and one unstable source b'^c_z with probability $1 - c^1_{b_z}$ where source source b'^1_z only emits perfect single-photon pulses and b'^c_z emits pulses in state $\rho^i_{b_c}$. With this, we have the following virtual protocol 2.

The virtual protocol 2

In the i-th time window, Alice (Bob) randomly decides it is a decoy window or a signal window with probabilities $1 - p_{a_z}$ and p_{a_z} ($1 - p_{b_z}$ and p_{b_z}). If it is a decoy window, Alice (Bob) randomly chooses the sources a_0, a'_1, a'_2 (b_0, b'_1, b'_2) with probabilities $p_{a_0} = 1 - p_{a_1} - p_{a_2}, p_{a_1}, p_{a_2}$ ($p_{b_0} = 1 - p_{b_1} - p_{b_2}, p_{b_1}, p_{b_2}$). If it is a signal window, Alice (Bob) randomly chooses the sources a_0, a'^1_z, a'^c_z (b_0, b'^1_z, b'^c_z) with probabilities $1 - \epsilon_a, \epsilon_a c^1_{a_z}, \epsilon_a(1 - c^1_{a_z})$ ($1 - \epsilon_b, \epsilon_b c^1_{b_z}, \epsilon_b(1 - c^1_{b_z})$), where the source a'^1_z only emits single-photon pulses and the source a'^c_z emits pulses in state $\rho^i_{a_c}$ (the source b'^1_z only emits single-photon pulses and the source b'^c_z emits pulses in state $\rho^i_{b_c}$), and if

$$\frac{\mu'^L_{a_z} e^{-\mu'^L_{a_z}}}{\mu'^L_{b_z} e^{-\mu'^L_{b_z}}} \leq \frac{\epsilon_b(1 - \epsilon_a)\mu^U_{a_1}}{\epsilon_a(1 - \epsilon_b)\mu^U_{b_1}}, \tag{12}$$

$c^1_{a_z}, c^1_{b_z}$ satisfy

$$c^1_{a_z} = \mu'^L_{a_z} e^{-\mu'^L_{a_z}}, \quad c^1_{b_z} = \frac{\epsilon_a(1 - \epsilon_b)\mu^U_{b_1}}{\epsilon_b(1 - \epsilon_a)\mu^U_{a_1}} c^1_{a_z}, \tag{13}$$

else

$$c^1_{b_z} = \mu'^L_{b_z} e^{-\mu'^L_{b_z}}, \quad c^1_{a_z} = \frac{\epsilon_b(1 - \epsilon_a)\mu^U_{a_1}}{\epsilon_a(1 - \epsilon_b)\mu^U_{b_1}} c^1_{b_z}. \tag{14}$$

The following precesses are the same with the virtual protocol 1. As discussed above, the virtual protocol 2 is equivalent to the virtual protocol 1. Eve can not distinguish the difference between the real protocol, virtual protocol 1 and virtual protocol 2.

We shall regard all those (single-photon) pulses from sources $\{a'^1_z, b_0\}$ and $\{a_0, b'^1_z\}$ in Z windows as untagged pulses. Clearly, a state of untagged pulse here is identical to the single-photon state in X windows due to

$$\frac{\mu^U_{a_1}}{\mu^U_{b_1}} = \frac{\epsilon_a(1 - \epsilon_b)c^1_{a_z}}{\epsilon_b(1 - \epsilon_a)c^1_{b_z}}. \tag{15}$$

Furthermore, only the sources a'_2 and b'_2 are unstable in virtual protocol 2. The remaining problems are estimating the lower bound of the number of untagged bits and the upper bound of the phase-flip error rate, both of which can be solved by directly applying the conclusion of Ref. [34]. Finally, using the method introduced in Ref. [28,29], we can obtain the final key rate. We can also use the AOPP method to improve the key rate [31,32]. The calculation details are shown in Sec. 1 of the supplement material.

Major results

1. Given the unstable sources as presented in the real protocol, we map them to virtual sources by virtual attenuation and tagged model, as shown in the virtual protocol 2.

2. In the virtual protocol 2, the source a_1' and b_1' are stable, with exactly known intensities, and the sources $a_z'^1$ and $b_z'^1$ are perfect single-photon sources, while other non-vacuum sources are unstable, with known bounds.

3. Regard the observed values in the real protocol to be those of the virtual protocol, and use decoy-state analysis to verify the value n_1^L and $e_1^{ph,U}$, which are the lower bound value of the number of untagged bits in Z windows and the upper bound value of the phase-flip error rate.

4. Calculate the key rate for the real protocol by the following formula:

$$R = \frac{1}{N}\{n_1^L[1 - H(e_1^{ph,U})] - fn_t H(E) - \log_2 \frac{2}{\varepsilon_{cor}} - 2\log_2 \frac{1}{\sqrt{2}\varepsilon_{PA}\hat{\varepsilon}}\}. \quad (16)$$

where N represents the total number of pulse pairs sent by Alice and Bob, f represents the error correction inefficiency, n_t represents the number of effective events in the Z windows, and E represents the error rate of the raw keys in the Z windows. As shown in Ref. [19,29], the tailing term of $-\log_2 \frac{2}{\varepsilon_{cor}} - 2\log_2 \frac{1}{\sqrt{2}\varepsilon_{PA}\hat{\varepsilon}}$ is the additional cost for security with finite size. The calculation details are shown in Sec. 1 of the supplement material. As detailed in the supplement material, our method does not presume the source errors to be random. Our protocol assumes that Eve can know or even determine the error values of all those candidate states in advance, so they surely are not limited to be random errors only. For security, the decoy state method requests that Eve has no information in advance about secret state choice of Alice and Bob, i.e., choosing the pulse of which source to send out by Alice and Bob for each time windows. To keep this condition, our method requests that the values of intensity errors which can be known to Eve in advance do not carry any private information of state choice of Alice and Bob. With this presumption being respected, our method allows whatever dependence of intensity errors from different time windows.

5. The AOPP method is directly applicable here. Suppose Bob generates the active odd-parity random pairing and then informs Alice of the positions of two bits in raw keys for each pair. Following the parity check, all pairs with odd-parity values at Alice's side survive, and one bit from each survived pair is chosen at random for final key distillation. That is, in the AOPP, a pair with two untagged bits will always contribute an untagged bit to the final key distillation. Those pairs with even parity values on Alice's side will be completely discarded. We have the following final key length formula after AOPP

$$R' = \frac{1}{N}\{n_1'^L[1 - H(e_1'^{ph,U})] - fn_t' H(E') - 2\log_2 \frac{2}{\varepsilon_{cor}} - 4\log_2 \frac{1}{\sqrt{2}\varepsilon_{PA}\hat{\varepsilon}}\}. \quad (17)$$

where $n_1'^L$ is the lower bound of untagged bits after AOPP; $e_1'^{ph,U}$ is the upper bound of the phase-flip error rate after AOPP; n_t' is the number of survived bits after AOPP; E' is the bit-flip error rate of the survived bits after AOPP. Details are shown in Sec. 1 of the supplement material.

Numerical simulation

We use the linear model to simulate the observed values [28], and Charlie's detectors are assumed to be identical, that is, they have the same detection efficiency and dark counting rate. For simplicity, we assume the maximum derivations of all sources are the same, i.e., $\delta_{a_l} = \delta_{b_r} = \delta$ for $l, r = 1, 2, z$. The experiment parameters are listed in Table. 1.

Figures 2 and 3 are the key rates of the original SNS protocol and the SNS protocol with the AOPP method under different degrees of intensity fluctuation. We assume a symmetric channel, which means that the distance between Alice and Charlie, L_{AC}, equals the distance between Bob and Charlie, L_{BC}. Figure 2 and 3 show that the key rates and farthest distance decrease slightly as the intensity fluctuation range increases. Figure 4 is the comparison of the key rates of the original SNS protocol and the SNS protocol with the AOPP method under different degrees of intensity fluctuation, where we set $L_{AC} - L_{BC}$ =50 km. The key rates at the distance of 350 km are shown in Table 3, where the experiment parameters are the same as those of Figure 4. In Figures 2-4, the absolute PLOB bound is for the case with perfect detection efficiency. Results in those Figures show that the key rates of our method can still exceed the absolute PLOB bound with 5% intensity fluctuation. As the intensity fluctuation increases, so do the distance of the first intersection between the absolute PLOB bound and the key rate curve, implying that it is more difficult to exceed the absolute PLOB bound with a larger intensity fluctua-

p_d	e_d	η_d	f	α_f	ξ	N
1.0×10^{-8}	4%	60.0%	1.1	0.2	1.0×10^{-10}	1.0×10^{13}

Table 1. List of experimental parameters used in numerical simulations. Here p_d is the dark counting rate per pulse of Charlie's detectors; e_d is the misalignment-error probability; η_d is the detection efficiency of Charlie's detectors; f is the error correction inefficiency; α_f is the fiber loss coefficient (dB/km); ξ is the failure probability while using Chernoff bound [37]; N is the number of total pulse pairs sent out in the protocol.

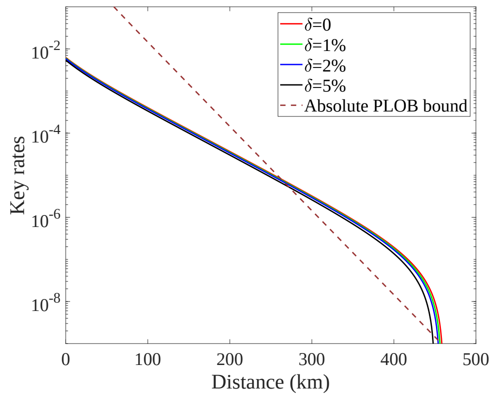

Figure 2. The the key rates of the original SNS protocol under different degrees of intensity fluctuation. The absolute PLOB bound is the PLOB bound with 100% detection efficiency detectors. Here we assume the symmetric channel, i.e., the distance between Alice and Charlie, L_{AC}, equals to the distance between Bob and Charlie, L_{BC}. The experiment parameters are listed in Table. 1.

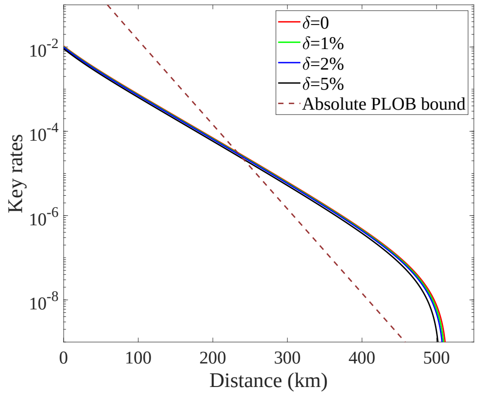

Figure 3. The the key rates of the SNS protocol with AOPP under different degrees of intensity fluctuation. The absolute PLOB bound is the PLOB bound with 100% detection efficiency detectors. Here we assume the symmetric channel, i.e., the distance between Alice and Charlie, L_{AC}, equals to the distance between Bob and Charlie, L_{BC}. The experiment parameters are listed in Table. 1.

tion, and this trend is more apparent in Figure 4. The key rates and distances corresponding to the first intersections in Figures 2-4 are shown in Table 2.

We use a more efficient method to calculate the key rates of the AOPP-SNS in Table 3, by scanning the expected value of the counting rate of vacuum sources $\langle S_{00}\rangle$ in its range, which is

$$R'' = \min_{\langle S_{00}\rangle} R'(\langle S_{00}\rangle), \tag{18}$$

and this can improve the non-asymptotic key rate a little bit. The results in Table 3 show that the key rates of AOPP with 5% intensity fluctuation are still higher than those of the original SNS protocol without intensity fluctuation. With Table 3, we can see that every 1% intensity fluctuation causes a 4% drop in key rate, which shows that the SNS protocol is robust against the source errors.

The AOPP method was used in the SNS experiment in 511 km field fiber to achieve a higher key rate [8]. We calculate the key rate of this experiment if there are source errors using the experiment observed values shown in Ref. [8], and the results show that the key rate is still positive even if δ is as large as 9.5%.

In the security proof and numerical simulation above, we assume the intensities of the

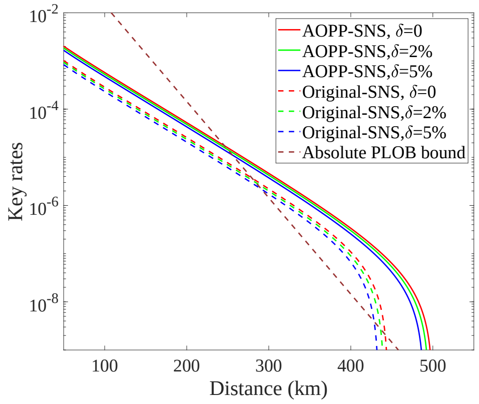

Figure 4. The comparison of the key rates of the original SNS protocol and the SNS protocol with AOPP method under different degrees of intensity fluctuation. The absolute PLOB bound is the PLOB bound with 100% detection efficiency detectors. Here we assume the asymmetric channel, where the distance between Alice and Charlie, L_{AC}, and the distance between Bob and Charlie, L_{BC}, satisfy $L_{AC}-L_{BC}$ =50 km. The experiment parameters are listed in Table. 1.

δ	0%	2%	5%	0%	2%	5%
Channel	symmetric	symmetric	symmetric	symmetric	symmetric	symmetric
Method	AOPP	AOPP	AOPP	Original	Original	Original
Distance	233	237	241	263	267	272
Key rate	3.16×10^{-5}	2.67×10^{-5}	2.18×10^{-5}	8.00×10^{-6}	6.66×10^{-6}	5.24×10^{-6}
Channel	asymmetric	asymmetric	asymmetric	asymmetric	asymmetric	asymmetric
Method	AOPP	AOPP	AOPP	Original	Original	Original
Distance	246	250	256	280	284	291
Key rate	1.73×10^{-5}	1.44×10^{-5}	1.11×10^{-5}	3.66×10^{-6}	3.00×10^{-6}	2.18×10^{-6}

Table 2. The key rates and distances corresponding to the first intersections between the absolute PLOB bound and the key rate curves in Figures 2-4. Here the results of the original method with symmetric channel are the intersections in Figure 2; the results of the AOPP method with symmetric channel are the intersections in Figure 3; the results of the AOPP and original methods with asymmetric channel are the intersections in Figure 4.

δ	0%	2%	5%	10%
Original-SNS	5.8×10^{-7}	5.15×10^{-7}	4.35×10^{-7}	3.26×10^{-7}
AOPP-SNS	1.33×10^{-6}	1.20×10^{-6}	1.05×10^{-6}	8.46×10^{-7}

Table 3. The key rates of the original SNS protocol and the AOPP method under different degrees of intensity fluctuation. The distance between Alice and Bob is 350 km, and the experiment parameters are the same with those of Figure. 4. In our simulation, the fluctuation parameter δ is a range between $-\delta$ and δ. For example, $\delta = 10\%$ means a fluctuating range of 20%, from −10% to +10%.

pulses are in a certain interval. But a more practical case is that very few pulses can exceed the bound values and we can only determine at most how many pulses outside the interval with a small failure probability. We show how to calculate the key rate under this case in Sec. 4 of the supplemental material.

Conclusion

In this study, we propose a strict method for calculating the key rate of the SNS protocol with source errors, by establishing the equivalent protocols through virtual attenuation and tagged model. We finally obtain the key rate formulas under the premise of ensuring the protocol's security. Our method can be combined with the AOPP method to further improve the key rate. The numerical results show that every 1% intensity fluctuation causes a 4% drop in key rate; additionally, the SNS protocol's farthest distance slightly decreases as the intensity fluctuation range increases, indicating that the SNS protocol is robust against the source errors. Our method can be directly applied to the 3-intensity SNS protocol with source error where '3-intensity' refers $\mu_{a_2} = \mu_{a_z}, \mu_{b_2} = \mu_{b_z}$.

Since our method on the one hand allows Eve to know the error values in advance, on the other hand requests no information leakage of state choice, the errors must be independent of state choice in applying our method [38]. With these conditions being respected, our method applies to errors inside the bound value with whatever patterns. Our method does not work with crossing correlations between intensity errors and state choice at different time windows, say, setting-choice-dependent errors [39–41], because such types of crossing correlations obviously lead to the state-choice information leakage to Eve if she knows the error values in advance. It is an interesting problem for future study on the setting-choice-dependent errors [39–41] with TF-QKD protocols.

Author contributions

Cong Jiang and Xiang-Bin Wang conceived the research and developed the security proof. Cong Jiang, Zong-Wen Yu and Xiao-Long Hu performed the numerical simulation. All authors performed the data analysis and prepared the manuscript.

Funding

We acknowledge the financial support in part by Ministration of Science and Technology of China through The National Key Research and Development Program of China Grant No. 2020YFA0309701; National Natural Science Foundation of China Grant Nos. 12174215, 12104184, 11774198 and 11974204; Shandong Provincial Natural Science Foundation Grant No. ZR2021LLZ007; Key R&D Plan of Shandong Province Grant No. 2021ZDPT01; Open Research Fund Program of the State Key Laboratory of Low-Dimensional Quantum Physics Grant No. KF202110; Leading Talents of Quancheng Industry.

Conflict of interest

None declared.

Data Availability.

The data that support the findings of this study are available from the corresponding author upon reasonable request.

REFERENCES

1. Lucamarini M, Yuan ZL, Dynes JF *et al.* Overcoming the rate–distance limit of quantum key distribution without quantum repeaters. *Nature* 2018; **557**: 400.
2. Liu Y, Yu ZW, Zhang W *et al.* Experimental twin-field quantum key distribution through sending or not sending. *Physical Review Letters* 2019; **123**: 100505.
3. Wang S, He DY, Yin ZQ *et al.* Beating the fundamental rate-distance limit in a proof-of-principle quantum key distribution system. *Physical Review X* 2019; **9**: 021046.
4. Zhong X, Hu J, Curty M *et al.* Proof-of-principle experimental demonstration of twin-field type quantum key distribution. *Physical Review Letter* 2019; **123**: 100506.
5. Fang XT, Zeng P, Liu H *et al.* Implementation of quantum key distribution surpassing the linear rate-transmittance bound. *Nature Photonics* 2020; **14**: 422–425.
6. Chen JP, Zhang C, Liu Y *et al.* Sending-or-not-sending with independent lasers: Secure twin-field quantum key distribution over 509 km. *Physical review letters* 2020; **124**: 070501.
7. Liu H, Jiang C, Zhu HT *et al.* Field test of twin-field quantum key distribution through sending-or-not-sending over 428 km. *Physical Review Letters* 2021; **126**: 250502.
8. Chen JP, Zhang C, Liu Y *et al.* Twin-field quantum key distribution over a 511 km optical fibre linking two distant metropolitan areas. *Nature Photonics* 2021; **15**: 570.
9. Pittaluga M, Minder M, Lucamarini M *et al.* 600 km repeater-like quantum communications with dual-band stabilisation. *Nature Photonics* 2021; **15**: 530.
10. Bennett CH and Brassard G. Quantum cryptography: Public key distribution and coin tossing. *Proceedings of the IEEE International Conference on Computers, Systems, and Signal Processing* (1984) 175–179.
11. Hwang WY. Quantum key distribution with high loss: toward global secure communication. *Physical Review Letters* 2003; **91**: 057901.
12. Wang XB. Beating the photon-number-splitting attack in practical quantum cryptography. *Physical Review Letters* 2005; **94**: 230503.
13. Lo HK, Ma X and Chen K. Decoy state quantum key distribution. *Physical Review Letters* 2005; **94**: 230504.
14. Lim CCW, Curty M, Walenta N *et al.* Concise security bounds for practical decoy-state quantum key distribution. *Physical Review A* 2014; **89**: 022307.
15. Boaron A, Boso G, Rusca D *et al.* Secure quantum key distribution over 421 km of optical fiber. *Physical Review Letters* 2018; **121**: 190502.
16. Lo HK, Curty M and Qi B. Measurement-device-independent quantum key distribution. *Physical Review Letters* 2012; **108**: 130503.
17. Braunstein SL and Pirandola S. Side-channel-free quantum key distribution. *Physical Review Letters* 2012; **108**: 130502.
18. Wang XB. Three-intensity decoy-state method for device-independent quantum key distribution with basis-dependent errors. *Physical Review A* 2013; **87**: 012320.
19. Curty M, Xu F, Cui W *et al.* Finite-key analysis for measurement-device-independent quantum key distribution. *Nature Communications* 2014; **5**: 4732.
20. Xu F, Xu H and Lo HK. Protocol choice and parameter optimization in decoy-state measurement-device-independent quantum key distribution. *Physical Review A* 2014; **89**: 052333.
21. Yu ZW, Zhou YH and Wang XB. Statistical fluctuation analysis for measurement-device-independent quantum key distribution with three-intensity decoy-state method. *Physical Review A* 2015; **91**: 032318.
22. Zhou YH, Yu ZW and Wang XB. Making the decoy-state measurement-device-independent quantum key distribution practically useful. *Physical Review A* 2016; **93**: 042324.
23. Pirandola S, Laurenza R, Ottaviani C *et al.* Fundamental limits of repeaterless quantum communications. *Nature Communications* 2017; **8**: 15043.
24. Wang XB, Yu ZW and Hu XL. Twin-field quantum key distribution with large misalignment error. *Physical Review A* 2018; **98**: 062323.
25. Tamaki K, Lo HK, Wang W *et al.* Information theoretic security of quantum key distribution overcoming the repeaterless secret key capacity bound. *arXiv preprint arXiv:1805.05511* 2018; .
26. Cui C, Yin ZQ, Wang R *et al.* Twin-field quantum key distribution without phase postselection. *Physical Review Applied* 2019; **11**: 034053.
27. Curty M, Azuma K and Lo HK. Simple security proof of twin-field type quantum key distribution protocol. *NPJ Quantum Information* 2019; **5**: 64.
28. Yu ZW, Hu XL, Jiang C *et al.* Sending-or-not-sending twin-field quantum key distribution in practice. *Scientific Reports* 2019; **9**: 3080.
29. Jiang C, Yu ZW, Hu XL *et al.* Unconditional security of sending or not sending twin-field quantum key distribution with finite pulses. *Physical Review Applied* 2019; **12**: 024061.
30. Hu XL, Jiang C, Yu ZW *et al.* Sending-or-not-sending twin-field protocol for quantum key distribution with asymmetric source parameters. *Physical Review A* 2019; **100**: 062337.
31. Xu H, Yu ZW, Jiang C *et al.* Sending-or-not-sending twin-field quantum key distribution: Breaking the direct transmission key rate. *Physical Review A* 2020; **101**: 042330.
32. Jiang C, Hu XL, Yu ZW *et al.* Composable security for practical quantum key distribution with two way classical communication. *New Journal of Physics* 2021; **23**: 063038.
33. Clivati C, Meda A, Donadello S *et al.* Coherent phase transfer for real-world twin-field quantum key distribution. *Nature Communications* 2022; **13**: 157.
34. Wang XB, Peng CZ, Zhang J *et al.* General theory of decoy-state quantum cryptography with source errors. *Physical Review A* 2008; **77**: 042311.
35. Jiang C, Yu ZW and Wang XB. Measurement-device-independent quantum key distribution with source state errors in photon number space. *Physical Review A* 2016; **94**: 062323.

36. Wang XB, Peng CZ and Pan JW. Simple protocol for secure decoy-state quantum key distribution with a loosely controlled source. *Applied Physics Letters* 2007; **90**: 031110.
37. Chernoff H. A measure of asymptotic efficiency for tests of a hypothesis based on the sum of observations. *The Annals of Mathematical Statistics* 1952; **23**: 493–507.
38. Mizutani A, Kato G, Azuma K *et al.* Quantum key distribution with setting-choice-independently correlated light sources. *npj Quantum Information* 2019; **5**: 1–8.
39. Pereira M, Kato G, Mizutani A *et al.* Quantum key distribution with correlated sources. *Science Advances* 2020; **6**: eaaz4487.
40. Zapatero V, Navarrete Á, Tamaki K *et al.* Security of quantum key distribution with intensity correlations. *Quantum* 2021; **5**: 602.
41. Sixto X, Zapatero V and Curty M. Security of decoy-state quantum key distribution with correlated intensity fluctuations. *arXiv preprint arXiv:2206.06700* 2022; .
