# Supplementary material for: Robust twin-field quantum key distribution through sending or not sending
Source: Natl Sci Rev. 2022 Sep 19;10(4):nwac186. doi: 10.1093/nsr/nwac186 (PMC10115169; doi:10.1093/nsr/nwac186)
Supplement: nwac186_Supplemental_File [file nwac186_supplemental_file.zip › SupplementalMaterials-red.pdf]

Supplemental Material

I. THE CALCULATION METHOD

Except the data of the Z windows, the other data are used to perform the decoy state analysis including the data when either or both of Alice and Bob choose the decoy windows. In the decoy-state analysis, we shall only focus on these data (all data except the data of the Z windows).

We simplify the symbols of two pulse sources $a_l, b_r (l, r = 0, 1, 2)$ as lr. We denote the number of pulse pairs of source lr sent out in the whole protocol by N_{lr}, and the total number of one-detector heralded events of source lr by n_{lr}, whose expected value is $\langle n_{lr}\rangle$. We define the counting rate of source lr by $S_{lr} = n_{lr}/N_{lr}$, and the corresponding expected value by $\langle S_{lr}\rangle$. The Chernoff bound can be used to estimate the lower and upper bound of the expected values according to their observed values. We have

$$\begin{aligned}
N_{00} =&[(1-p_{a_z})(1-p_{b_z})p_{a_0}p_{b_0} + (1-p_{a_z})p_{b_z}p_{a_0}(1-\epsilon_b) \\
&+ p_{a_z}(1-p_{b_z})(1-\epsilon_a)p_{b_0}]N, \\
N_{01} =&[(1-p_{a_z})p_{a_0} + p_{a_z}(1-\epsilon_a)](1-p_{b_z})p_{b_1}N, \\
N_{10} =&[(1-p_{b_z})p_{b_0} + p_{b_z}(1-\epsilon_b)](1-p_{a_z})p_{a_1}N, \\
N_{02} =&[(1-p_{a_z})p_{a_0} + p_{a_z}(1-\epsilon_a)](1-p_{b_z})p_{b_2}N, \\
N_{20} =&[(1-p_{b_z})p_{b_0} + p_{b_z}(1-\epsilon_b)](1-p_{a_z})p_{a_2}N.
\end{aligned} \tag{1}$$

We denote the number of pulse pairs of the X windows sent out in the whole protocol by N_X, and the number of effective wrong events by M_X, whose expected value is $\langle M_X\rangle$. We define $T_X = M_X/N_X$, and its expected value by $\langle T_X\rangle$.

Since the sources are unstable, we can not directly apply the results of traditional decoy-state method [1–3]. As the discussion in the main body, we can take the virtual protocol 2 to calculate the final key rate according to the observed values in the real protocol. The density matrices of the stable sources a'_1 and b'_1 are

$$\begin{aligned}
\rho_{a'_1} &= \sum_{k=0}^{+\infty} c_{a_1}^k |k\rangle\langle k|, \quad c_{a_1}^k = \frac{(\mu_{a_1}^U)^k e^{-\mu_{a_1}^U}}{k!}, \\
\rho_{b'_1} &= \sum_{k=0}^{+\infty} c_{b_1}^k |k\rangle\langle k|, \quad c_{b_1}^k = \frac{(\mu_{b_1}^U)^k e^{-\mu_{b_1}^U}}{k!}.
\end{aligned} \tag{2}$$

For the unstable sources a'_2 and b'_2, the density matrices of the pulses in the $i-$th time window are

$$\begin{aligned}
\rho_{a'_2}^i &= \sum_{k=0}^{+\infty} c_{a_2}^{k,i} |k\rangle\langle k|, \quad c_{a_2}^{k,i} = \frac{(\mu_{a_2}^{\prime i})^k e^{-\mu_{a_2}^{\prime i}}}{k!}, \\
\rho_{b'_2}^i &= \sum_{k=0}^{+\infty} c_{b_2}^{k,i} |k\rangle\langle k|, \quad c_{b_2}^{k,i} = \frac{(\mu_{b_2}^{\prime i})^k e^{-\mu_{b_2}^{\prime i}}}{k!}.
\end{aligned} \tag{3}$$

For the stable two pulse sources, we denote the expected values of the counting rate of the state $|01\rangle\langle 01|$ and $|10\rangle\langle 10|$ as $\langle s_{01}\rangle$ and $\langle s_{10}\rangle$. We have [4]

$$\langle s_{01}\rangle \geq \langle s_{01}\rangle^L = \frac{c_{b_2}^{2,L}\langle S_{01}\rangle^L - c_{b_1}^2\langle S_{02}\rangle^U - (c_{b_1}^0 c_{b_2}^{2,L} - c_{b_1}^2 c_{b_2}^{0,L})\langle S_{00}\rangle^U}{c_{b_1}^1 c_{b_2}^{2,L} - c_{b_1}^2 c_{b_2}^{1,L}}, \tag{4}$$

and

$$\langle s_{10}\rangle \geq \langle s_{10}\rangle^L = \frac{c_{a_2}^{2,L}\langle S_{10}\rangle^L - c_{a_1}^2\langle S_{20}\rangle^U - (c_{a_1}^0 c_{a_2}^{2,L} - c_{a_1}^2 c_{a_2}^{0,L})\langle S_{00}\rangle^U}{c_{a_1}^1 c_{a_2}^{2,L} - c_{a_1}^2 c_{a_2}^{1,L}}. \tag{5}$$

If Bob does the error correction computation and he chooses to take decoy-state analysis after error correction, he can verify the bounds of $\langle S_{00}\rangle$ more tightly by using the events of all those time windows a_0b_0, i.e., when both parties used vacuum source, because he knows all of them after error correction. Say, after error correction, for all those survived pairs in AOPP, he knows which bits are from time windows of a_0b_0, while for those rejected pairs in AOPP and all those time windows without correct heralding at Charlie's measurement station, they can publicly announce the corresponding bit values. In this way Bob can simply use $n_{a_0b_0}/N_{a_0b_0}$ for his observed value of $\langle S_{00}\rangle$,

and hence the protocol does not need to reserve any vacuum windows as random samples to test S_{00}. This improves the non-asymptotic key rate a little bit because we can verify $\langle S_{00}\rangle$ more efficiently.

We denote the expected values of the counting rate of the untagged pulses as $\langle s_1\rangle$, and we have [3]

$$\langle s_1\rangle \ge \langle s_1\rangle^L = \frac{\mu_{a_1}^U}{\mu_{a_1}^U+\mu_{b_1}^U}\langle s_{10}\rangle^L + \frac{\mu_{b_1}^U}{\mu_{a_1}^U+\mu_{b_1}^U}\langle s_{01}\rangle^L. \tag{6}$$

The upper bound of the expected value of the phase-flip error rate is

$$\langle e_1^{ph}\rangle \le \langle e_1^{ph}\rangle^U = \frac{\langle T_X\rangle^U - e^{-\mu_{a_1}^U-\mu_{b_1}^U}\langle S_{00}\rangle^L/2}{e^{-\mu_{a_1}^U-\mu_{b_1}^U}(\mu_{a_1}^U+\mu_{b_1}^U)\langle s_1\rangle^L}. \tag{7}$$

Then we have the lower bound of the real value of the untagged bits in the Z windows

$$n_1^L = O^L(\langle n_1\rangle^L) \tag{8}$$

where

$$\langle n_1\rangle^L = Np_{a_z}p_{b_z}[\epsilon_a(1-\epsilon_b)c_{a_z}^1 + \epsilon_b(1-\epsilon_a)c_{b_z}^1]\langle s_1\rangle^L, \tag{9}$$

and $O^L(Y)$ is defined in Eq. (20). And the upper bound of the real value of the phase-flip error rate is

$$e_1^{ph,U} = \frac{O^U(n_1^L\langle e_1^{ph}\rangle^U)}{n_1^L}. \tag{10}$$

Finally, we get the secure final key rate

$$R = \frac{1}{N}\left\{n_1^L[1-H(e_1^{ph,U})] - fn_tH(E) - \log_2\frac{2}{\varepsilon_{cor}} - 2\log_2\frac{1}{\sqrt{2}\varepsilon_{PA}\hat{\varepsilon}}\right\}. \tag{11}$$

We set $\varepsilon_{cor}=\varepsilon_{PA}=\hat{\varepsilon}=10^{-10}$ in this article.

Besides, with all those data, we can use the AOPP method shown in Ref. [5] to get higher key rate. With values above, we can calculate the lower bound of untagged bits and phase-flip error rate after AOPP, $n_1'^L$ and $e_1'^{ph,U}$ by the method proposed in Refs. [5, 6]. We have the related formulas of $n_1'^{,L}$ as follows:

$$\langle n_{u1}\rangle^L = Np_{a_z}p_{b_z}\epsilon_a(1-\epsilon_b)c_{a_z}^1\langle s_{10}\rangle^L, \tag{12a}$$

$$\langle n_{u0}\rangle^L = Np_{a_z}p_{b_z}\epsilon_b(1-\epsilon_a)c_{b_z}^1\langle s_{01}\rangle^L, \tag{12b}$$

$$u = \frac{n_g}{2n_{odd}}, \tag{12c}$$

$$n_{u1}^L = \varphi^L(u\langle n_{u1}\rangle^L), \tag{12d}$$

$$n_{u0}^L = \varphi^L(u\langle n_{u0}\rangle^L), \tag{12e}$$

$$n_1^L = n_{u1}^L + n_{u0}^L, \tag{12f}$$

$$n_1^r = \varphi^L\left(\frac{(n_1^L)^2}{2un_t}\right), \tag{12g}$$

$$n_{u1}' = 2n_1^r\left(\frac{n_{u1}^L}{n_1^L} - \sqrt{-\frac{\ln\varepsilon}{2n_1^r}}\right) \tag{12h}$$

$$n_{u0}' = 2n_1^r\left(\frac{n_{u0}^L}{n_1^L} - \sqrt{-\frac{\ln\varepsilon}{2n_1^r}}\right) \tag{12i}$$

$$n_{min} = \min(n_{01}', n_{10}'), \tag{12j}$$

$$n_1'^{,L} = 2\varphi^L\left(n_{min}(1-\frac{n_{min}}{2n_1^r})\right), \tag{12k}$$

where n_g is the number of pair if Alice and Bob perform AOPP to their raw keys; n_{odd} is the number of pairs with odd-parity if Bob randomly groups all the raw keys two by two, and n_g and n_{odd} are observed values; ε is the failure

probability of parameter estimation; and $\varphi^U(x), \varphi^L(x)$ are the upper and lower bounds while using Chernoff bound [7] to estimate the real values according to the expected values, whose details are shown in Sec. II.

And we have the the related formulas of $e_1^{\prime ph}$ as follows:

$$r = \frac{n_1^L}{n_1^L - 2n_1^r} \ln \frac{3(n_1^L - 2n_1^r)^2}{\varepsilon}, \tag{13a}$$

$$e_\tau = \frac{\varphi^U(2n_1^r \langle e_1^{ph} \rangle^U)}{2n_1^r - r} \tag{13b}$$

$$M_s^U = \varphi^U[(n_1^r - r)e_\tau(1 - e_\tau)] + r, \tag{13c}$$

$$e_1^{\prime ph,U} = \frac{2M_s^U}{n_1^\prime} \tag{13d}$$

Finally, we have the following final key length formula after AOPP

$$R' = \frac{1}{N}\left\{ n_1^{\prime L}[1 - H(e_1^{\prime ph,U})] - f n_t^\prime H(E') - 2\log_2 \frac{2}{\varepsilon_{cor}} - 4\log_2 \frac{1}{\sqrt{2}\varepsilon_{PA}\hat{\varepsilon}} \right\}. \tag{14}$$

II. CHERNOFF BOUND

The Chernoff bound can help us estimate the expected value from their observed values [7]. Let $X_1, X_2, \ldots, X_n$ be n independent random samples, detected with the value 1 or 0, and let X denote their sum satisfying $X = \sum_{i=1}^n X_i$. E is the expected value of X. We have

$$E^L(X) = \frac{X}{1 + \delta_1(X)}, \tag{15}$$

$$E^U(X) = \frac{X}{1 - \delta_2(X)}, \tag{16}$$

where we can obtain the values of $\delta_1(X)$ and $\delta_2(X)$ by solving the following equations

$$\left(\frac{e^{\delta_1}}{(1+\delta_1)^{1+\delta_1}} \right)^{\frac{X}{1+\delta_1}} = \xi, \tag{17}$$

$$\left(\frac{e^{-\delta_2}}{(1-\delta_2)^{1-\delta_2}} \right)^{\frac{X}{1-\delta_2}} = \xi, \tag{18}$$

where ξ is the failure probability.

Besides, we can use the Chernoff bound to help us estimate their real values from their expected values. Similar to Eqs. (15)- (18), the observed value, O, and its expected value, Y, satisfy

$$O^U(Y) = [1 + \delta_1'(Y)]Y, \tag{19}$$

$$O^L(Y) = [1 - \delta_2'(Y)]Y, \tag{20}$$

where we can obtain the values of $\delta_1'(Y, \xi)$ and $\delta_2'(Y, \xi)$ by solving the following equations

$$\left(\frac{e^{\delta_1'}}{(1+\delta_1')^{1+\delta_1'}} \right)^{Y} = \xi, \tag{21}$$

$$\left(\frac{e^{-\delta_2'}}{(1-\delta_2')^{1-\delta_2'}} \right)^{Y} = \xi. \tag{22}$$

III. SOME REMARKS OF OUR PROTOCOL AND APPLICABILITY OF CHERNOFF BOUND

In applying our method, we don't request the values of intensities (or intensity errors) to be random, there can be patterns among those data. There are two important points here: First, Alice and Bob does not need to know the values of intensities; second, we assume that Eve can know the values in advance.

Our method can be efficiently understood in this way: the intensity values of candidate pulses can be predetermined by any third party including Eve in whatever way provided that in predetermining them, he or she has no information of the secret state choice taken by Alice and Bob in the experiment, e.g., choosing which state to send out at each time windows [8]. In predetermining those explicit values of intensities (or intensity errors), the third party does not have to generate them randomly. He (or she) can take whatever way, e.g., he (or she) can first generate the values of a few time windows and then based on these values to determine all values by using certain functional. The condition of "no information of the secret state choice taken by Alice and Bob" for the third party only forbids the crossing correlation among intensity values and the secret state choice taken by Alice and Bob [8], but it does not forbid other types of dependence of intensity values among different time windows.

We use the fact that there exist explicit values of intensity errors in a real experiment. If one regard this as a "condition" rather than a "fact", then this condition actually always hold in any real experiment. If Eve knows those intensity values explicitly, our virtual protocols with attenuations hold exactly and hence the result of our real protocol is secure, as shown in the text. If Eve does not know those values explicitly, the result of our real protocol must be also secure because a weakened Eve who does not know those values explicitly cannot be more powerful in attacking the real protocol than an unweakened Eve who knows them explicitly. In particular, our result holds even though the third party predetermines the intensity errors in a certain probabilistic distribution of many sets of values of intensity errors of different time windows rather than one set of deterministic values. Because such a case simply means that the intensity values could be set 1, could be set 2, and so on. No matter which set it actually is, Alice and Bob can go ahead to obtain the their final bits by the real protocol because they do not need the explicit values in carrying out the real protocol. On the other hand, the final bits obtained by the real protocol must be also secure. Again, this is because a weakened Eve who does not know explicitly which set of intensity values in the experiment cannot be more powerful than the Eve who knows it explicitly.

In this paper, we apply the decoy-state analysis proposed in Ref. [4] to estimate the parameters of SNS protocol. To show that the Chernoff bound can be applied here, we take the decoy-state polarization BB84 protocol as an example, where Alice take decoy-state method with 3 sources which are the vacuum source o, the decoy source x and the signal source y. Given discussions above, we can now do the calculation with the following model for source intensity errors:

We assume that all values of intensities (or intensity errors) are in a known range [4]. There are no crossing independence between intensity values and the secret information of state choice [8]. With these conditions, we take the worst-case that Eve knows in advance the intensity errors explicitly of each candidate pulses. In our study, we use constant probabilities p_o, p_x, p_y for Alice to choose the vacuum source o, the decoy source x, and the signal source y at each time windows. Equivalently, we can use the following product state [8] for the source in our model:

$$\rho_{AB} = \otimes_{i=1}^{N}(p_o\rho_o \otimes |o_i\rangle\langle o_i|_A + p_x\rho_{\mu_{x_i}} \otimes |x_i\rangle\langle x_i|_A + p_y\rho_{\mu_{y_i}} \otimes |y_i\rangle\langle y_i|_A) \tag{23}$$

where the subscript A represents Alice's classical memories of storing which source are chosen, the states $\rho_o, \rho_{\mu_{x_i}}, \rho_{\mu_{y_i}}$ are send out to the channel. We have

$$\rho_o = \sum_{k=0} a_{o_i}^k |k\rangle\langle k|, \quad a_{o_i}^k = \delta_{0,k}, \tag{24}$$

$$\rho_{\mu_{x_i}} \sum_{k=0} a_{x_i}^k |k\rangle\langle k|, \quad a_{x_i}^k = \frac{(\mu_x^i)^k e^{-\mu_x^i}}{k!}, \tag{25}$$

$$\rho_{\mu_{y_i}} \sum_{k=0} a_{y_i}^k |k\rangle\langle k|, \quad a_{y_i}^k = \frac{(\mu_y^i)^k e^{-\mu_y^i}}{k!}. \tag{26}$$

We assume $\mu_x^L \leq \mu_x^i \leq \mu_x^U, \mu_y^L \leq \mu_y^i \leq \mu_y^U$ and $\mu_x^L, \mu_x^U, \mu_y^L, \mu_y^U$ are known values. This assumption is the same as that of Ref. [4].

With Eqs.(24-26), we can rewrite ρ_{AB} as the following form

$$\rho_{AB} = \otimes_{i=1}^{N}\left[\sum_{k=0}\left(p_o a_{o_i}^k |o_i\rangle\langle o_i|_A + p_x a_{x_i}^k |x_i\rangle\langle x_i|_A + p_y a_{y_i}^k |y_i\rangle\langle y_i|_A\right) \otimes |k\rangle\langle k|\right]. \tag{27}$$

Eq. (27) shows that the actual state for the sent-out pulse of time window i is simply an eigenstate in Fock space, with a classical probability for different photon numbers. Thus we can always regards that the actual state for the pulse to be sent out in the i-th time window is in a certain photon-number state $|m_i\rangle\langle m_i|$. Similar idea is also used in the original decoy-state method with exact intensities. At any time, the source emits a phase randomized coherent state which can be written in the probabilistic mixture of different photon-number state. We imagine that it actually emits

a Fock state at each time window, with the specific photon number being chosen by the corresponding probability distribution. Thus we can take the state prepared by Alice as

$$\tilde{\rho}_{AB} = \otimes_{i=1}^{N} \left[\left(p_o a_{o_i}^{m_i} |o_i\rangle\langle o_i|_A + p_x a_{x_i}^{m_i} |x_i\rangle\langle x_i|_A + p_y a_{y_i}^{m_i} |y_i\rangle\langle y_i|_A \right) \otimes |m_i\rangle\langle m_i| \right]. \tag{28}$$

This is a product state of Alice's local state and set-out state as following:

$$\tilde{\rho}_{AB} = \rho_{\mathcal{L}} \otimes \rho_{\mathcal{T}}. \tag{29}$$

Here either the local state $\rho_{\mathcal{L}}$ or the sent-out state $\rho_{\mathcal{T}}$ itself is a product state of different time windows:

$$\rho_{\mathcal{L}} = \otimes_{i=1}^{N} \left(p_o a_{o_i}^{m_i} |o_i\rangle\langle o_i|_A + p_x a_{x_i}^{m_i} |x_i\rangle\langle x_i|_A + p_y a_{y_i}^{m_i} |y_i\rangle\langle y_i|_A \right), \tag{30}$$

$$\rho_{\mathcal{T}} = \otimes_{i=1}^{N} |m_i\rangle\langle m_i| \tag{31}$$

Surely, any Eve's attack, including the attacks to the sent-out pulses can never change the local state $\rho_{\mathcal{L}}$, if Eve has no access to Alice's lab. If the pulse in i-th window causes a click, Alice can measure her local state to determine which source the pulse belongs to, and the probability that it is from source $l, l = o, x, y$ is

$$p_{i,l}^{m_i} = \frac{p_l a_{l_i}^{m_i}}{p_o a_{o_i}^{m_i} + p_x a_{x_i}^{m_i} + p_y a_{y_i}^{m_i}}. \tag{32}$$

Given the product form of local state shown in Eq. (30), observing $\rho_{\mathcal{L}_i} = p_o a_{o_i}^{m_i} |o_i\rangle\langle o_i|_A + p_x a_{x_i}^{m_i} |x_i\rangle\langle x_i|_A + p_y a_{y_i}^{m_i} |y_i\rangle\langle y_i|_A$ does not affect any state $\rho_{\mathcal{L}_j} = p_o a_{o_j}^{m_j} |o_j\rangle\langle o_j|_A + p_x a_{x_j}^{m_j} |x_j\rangle\langle x_j|_A + p_y a_{y_j}^{m_j} |y_j\rangle\langle y_j|_A$, provided that $i \neq j$. Thus we have *Fact C1*: values of $p_{i,l}^{m_i}$ of different time windows are *independent*.

Similar to Eq. (33) in Ref. [4], we have the following asymptotic formulas for the number of counts caused by each kind of pulses

$$\langle n_k^l \rangle = \sum_{i \in c_k} p_{i,l}^k = \sum_{i \in c_k} \frac{p_l a_{l_i}^k}{p_o a_{o_i}^k + p_x a_{x_i}^k + p_y a_{y_i}^k}, \tag{33}$$

where $\langle n_k^l \rangle$ is the expected value of the counts cased by the k-photon state from source l, and c_k is the set of all windows that cause counts by k-photon state pulses. We also have the expected value of all counts cased by source l

$$\langle N_l \rangle = \sum_{k=0} \langle n_k^l \rangle = \sum_{k=0} \sum_{i \in c_k} p_{i,l}^k. \tag{34}$$

Since all $p_{i,l}^k$ are independent, we can apply the Chernoff bound to estimate the bound values of $\langle N_l \rangle$ according to its corresponding observed values.

With Eq. (34) for $l = o, x, y$, we can get the lower bound of $\langle n_1^y \rangle$ [4]

$$\langle n_1^y \rangle \geq \langle n_1^y \rangle^L = \frac{a_y^{1,L} \left[a_y^{2,L} \langle N_x \rangle^L - a_x^{2,U} \langle N_y \rangle^U - (a_y^{2,L} a_x^{0,U} - a_x^{2,U} a_y^{0,L}) \langle N_0 \rangle^U p_y / p_o \right]}{a_x^{1,U} a_y^{2,L} - a_y^{1,L} a_x^{2,U}}, \tag{35}$$

where the subscript U, L represent the upper and lower bounds respectively.

Recall the express form of $\langle n_1^y \rangle$,

$$\langle n_1^y \rangle = \sum_{i \in c_1} p_{i,y}^1. \tag{36}$$

According to *Fact C1* above, all values $p_{i,y}^1$ of different i in the summation above are independent, and hence we can use the Chernoff bound to estimate the lower bound of n_1^y according to $\langle n_1^y \rangle$, where n_1^y is the real value of the counts cased by the single-photon state from source y. We have

$$n_1^y \geq O^L(\langle n_1^y \rangle), \tag{37}$$

except for a failure probability ξ, where $O^L(Y)$ is defined in Eq. (20). $O^L(Y)$ is a an increasing function of Y, which means if $Y_1 \leq Y_2$, then $O^L(Y_1) \leq O^L(Y_2)$. Thus we have

$$n_1^y \geq n_1^{y,L}, \tag{38}$$

except for a failure probability 4ξ, where

$$n_1^{y,L} = O^L(\langle n_1^y\rangle^L). \tag{39}$$

Above we have shown the worst-case result for the deterministic intensities of source pulses. Mathematically, this means

$$\Pr(n_1^y \geq n_1^{y,L}|\rho_D) \leq 4\xi, \tag{40}$$

where ρ_D is classical memories that store the intensities of all pulse. Since we only use the intensity error range in the whole precess, the result obviously holds for the case of probabilistic intensities given the same intensity error range, which is saying

$$\Pr(n_1^y \geq n_1^{y,L}) = \sum_{\rho_D} \Pr(n_1^y \geq n_1^{y,L}|\rho_D)\Pr(\rho_D) \leq 4\xi. \tag{41}$$

Above we have demonstrated the applicability of Chernoff bound through the decoy-state BB84 as an example. The same conclusion on the applicability obviously holds for SNS protocol which has been calculated in our main text.

IV. CALCULATION FORMULAS WITH SNS PROTOCOL

In the main text, we assume the intensities of the pulses are in a certain interval, $[(1-\delta)\mu, [(1+\delta)\mu]$. For a more practical case that we can only determine this interval probabilistically with a certain degree of confidence [8], we can use the following method to calculate the key rate.

In the real protocol, denote $\mathcal{C}^I$ as a set containing all time windows in which the intensities of all candidate states are in a certain interval defined in Eq.(1) in the main text. Denote $\mathcal{C}^O$ as a set containing all time windows in which at least on candidate state's intensity is out of the certain intervals. Apparently, we can apply the virtual attenuation method proposed in the main text to the pulse pairs in $\mathcal{C}^I$ and we get the virtual protocol. As for the pulse pairs in $\mathcal{C}^O$, they are the same in the virtual protocol and the real protocol.

In the virtual protocol, we have

$$\rho^i_{a'_l} = \sum_{k=0}^{+\infty} c^{k,i}_{a_l}|k\rangle\langle k|, \quad \rho^i_{b'_r} = \sum_{k=0}^{+\infty} c^{k,i}_{b_r}|k\rangle\langle k|, \tag{42}$$

for $l, r = 0, 1, 2, z$.

Let set c_k contains all $|k0\rangle$-photon-pair one-detector heralded events caused by sources $(a'_0b'_0, a'_1b'_0, a'_2b'_0, a'_zb'_0)$. Let set c^I_k be a subset of c_k that contains all one-detector heralded events caused by pulse pairs in time windows of $\mathcal{C}^I$, and let set c^O_k be a subset of c_k that contains all one-detector heralded event caused by pulse pairs in time windows of $\mathcal{C}^O$. We have

$$\begin{aligned}\langle n_{00}\rangle &= \sum_{i\in c_0}[p_{a_0}p_{b_0} + p_{a_0}p_{b_z}(1-\epsilon_b) + p_{a_z}(1-\epsilon_a)p_{b_0}]D^i_0 \\ &= \sum_{i\in c^I_0}[p_{a_0}p_{b_0} + p_{a_0}p_{b_z}(1-\epsilon_b) + p_{a_z}(1-\epsilon_a)p_{b_0}]D^i_0 + \Delta_0,\end{aligned} \tag{43}$$

$$\begin{aligned}\langle n_{10}\rangle &= \sum_{k=0}\sum_{i\in c_k} p_{a_1}[p_{b_0} + p_{b_z}(1-\epsilon_b)]c^{k,i}_{a_1}D^i_k \\ &= \sum_{k=0}\sum_{i\in c^I_k} p_{a_1}[p_{b_0} + p_{b_z}(1-\epsilon_b)]c^{k,i}_{a_1}D^i_k + \Delta_1,\end{aligned} \tag{44}$$

$$\begin{aligned}\langle n_{20}\rangle &= \sum_{k=0}\sum_{i\in c_k} p_{a_2}[p_{b_0} + p_{b_z}(1-\epsilon_b)]c^{k,i}_{a_2}D^i_k \\ &= \sum_{k=0}\sum_{i\in c^I_k} p_{a_2}[p_{b_0} + p_{b_z}(1-\epsilon_b)]c^{k,i}_{a_2}D^i_k + \Delta_2,\end{aligned} \tag{45}$$

where

$$D^i_k = \frac{1}{[p_{b_0} + p_{b_z}(1-\epsilon_b)]\{[p_{a_0} + p_{a_z}(1-\epsilon_a)]c^{k,i}_{a_0} + p_{a_1}c^{k,i}_{a_1} + p_{a_2}c^{k,i}_{a_2} + p_{a_z}\epsilon_a c^{k,i}_{a_z}\}}. \tag{46}$$

In this case, we determine the untagged bits only from the sources $a_0 b_z'^1$ and $a_z'^1 b_0$ of Z windows in $\mathcal{C}^I$, so we have

$$\langle n_{u1}\rangle = \sum_{i\in c_1^I} p_{a_z}\epsilon_a p_{b_z}(1-\epsilon_b)c_{a_z}^1 D_1^i. \tag{47}$$

Denote there are n_Δ elements in set $\mathcal{C}^O$. Similar to Ref. [8], we assume n_Δ is upper bounded by N_Δ with a failure probability at most ε_{fail}. Using the fact that there are at most N_Δ one-detector heralded event caused by pulse pairs in time windows of $\mathcal{C}^O$, we have

$$0 \le \Delta_j \le N_\Delta, \quad j = 0, 1, 2. \tag{48}$$

Combining Eqs.(43-48), we have

$$\langle n_{u1}\rangle^L = N p_{a_z} p_{b_z}\epsilon_a(1-\epsilon_b)c_{a_z}^1\langle s_{10}\rangle^{\prime,L}, \tag{49}$$

where

$$\langle s_{10}\rangle^{\prime,L} = \frac{c_{a_2}^{2,L}[\langle S_{10}\rangle^L - N_\Delta/N_{10}] - c_{a_1}^2\langle S_{20}\rangle^U - (c_{a_1}^0 c_{a_2}^{2,L} - c_{a_1}^2 c_{a_2}^{0,L})\langle S_{00}\rangle^U}{c_{a_1}^1 c_{a_2}^{2,L} - c_{a_1}^2 c_{a_2}^{1,L}}. \tag{50}$$

Similarly, we can get the formulas of $\langle n_{u0}\rangle^L$ and $\langle s_{01}\rangle^{\prime,L}$.

Define

$$\langle s_1\rangle^{\prime,L} = \frac{\mu_{a_1}^U}{\mu_{a_1}^U + \mu_{b_1}^U}\langle s_{10}\rangle^{\prime,L} + \frac{\mu_{b_1}^U}{\mu_{a_1}^U + \mu_{b_1}^U}\langle s_{01}\rangle^{\prime,L}, \tag{51}$$

we have the lower bound of the expected value of untagged bits

$$\langle n_1\rangle^L = N p_{a_z} p_{b_z}[\epsilon_b(1-\epsilon_a)c_{b_z}^1 + \epsilon_a(1-\epsilon_b)c_{b_z}^1]\langle s_1\rangle^{\prime,L}. \tag{52}$$

Denote the number of wrong untagged bits in the X windows of $\mathcal{C}^I$ by m_1, whose corresponding expected value is $\langle m_1\rangle$, we have $m_1 \le M_X$. The upper bound of the expected value of phase flip error rate of the untagged bits in the Z windows of $\mathcal{C}^I$ satisfy

$$\langle e_1^{ph}\rangle^U = \frac{\langle T_X\rangle^U}{e^{-\mu_{a_1}^U - \mu_{b_1}^U}(\mu_{a_1}^U + \mu_{b_1}^U)\langle s_1\rangle^{\prime,L}}. \tag{53}$$

Here we use the fact that the expected values of the untagged bits in the X windows and Z windows are proportional to their corresponding sending probabilities in the virtual protocol.

With all those values, we can calculate the final key rate of the SNS protocol. The failure probability ε_{fail} to estimate the upper bound of N_Δ contributes to the failure probability of parameter estimation. The only differences of the formulas here and those in Sec. I are the extra N_Δ/N_{10} in Eq. (50) and taking the lower bounds of vacuum counts as 0 in Eq. (53). In the typical experiments of TF-QKD, the counting rate of vacuum pulses is about 10^{-8} or smaller, and taking it as 0 has little effect on the key rate. Thus when N_Δ is small, for example $N_\Delta \sim 100$, we expect the method in this section would just affect the key rates a little.

Apparently, we can introduce virtual local state similar to Eq. (28) in deriving Eqs. (43-47). This means that quantities inside the summation of Eqs. (43-47) are independent to each other and hence the Chernoff bound applies in the calculation with finite data size.

[1] X.-B. Wang, Physical Review Letters **94**, 230503 (2005).
[2] Z.-W. Yu, X.-L. Hu, C. Jiang, H. Xu, and X.-B. Wang, Scientific Reports **9**, 3080 (2019).
[3] X.-L. Hu, C. Jiang, Z.-W. Yu, and X.-B. Wang, Physical Review A **100**, 062337 (2019).
[4] X.-B. Wang, C.-Z. Peng, J. Zhang, L. Yang, and J.-W. Pan, Physical Review A **77**, 042311 (2008).
[5] C. Jiang, X.-L. Hu, Z.-W. Yu, and X.-B. Wang, New Journal of Physics **23**, 063038 (2021).
[6] C. Jiang, X.-L. Hu, H. Xu, Z.-W. Yu, and X.-B. Wang, New Journal of Physics **22**, 053048 (2020).
[7] H. Chernoff, The Annals of Mathematical Statistics **23**, 493 (1952).
[8] A. Mizutani, G. Kato, K. Azuma, M. Curty, R. Ikuta, T. Yamamoto, N. Imoto, H.-K. Lo, and K. Tamaki, npj Quantum Information **5**, 1 (2019).
